# Supplementary material for: Vaccination against helminth IL-33 modulators permits immune-mediated parasite ejection
Source: Cell Rep. Author manuscript; Available in PMC 2026 Aug 15. (PMC7619366; doi:10.1016/j.celrep.2025.115721)
Supplement: Supplementary file 1 [file EMS217148-supplement-Document_S1__Figures_S1_S8_.pdf]

Cell Reports

Article

Vaccination against helminth IL-33 modulators permits immune-mediated parasite ejection

Graphical abstract

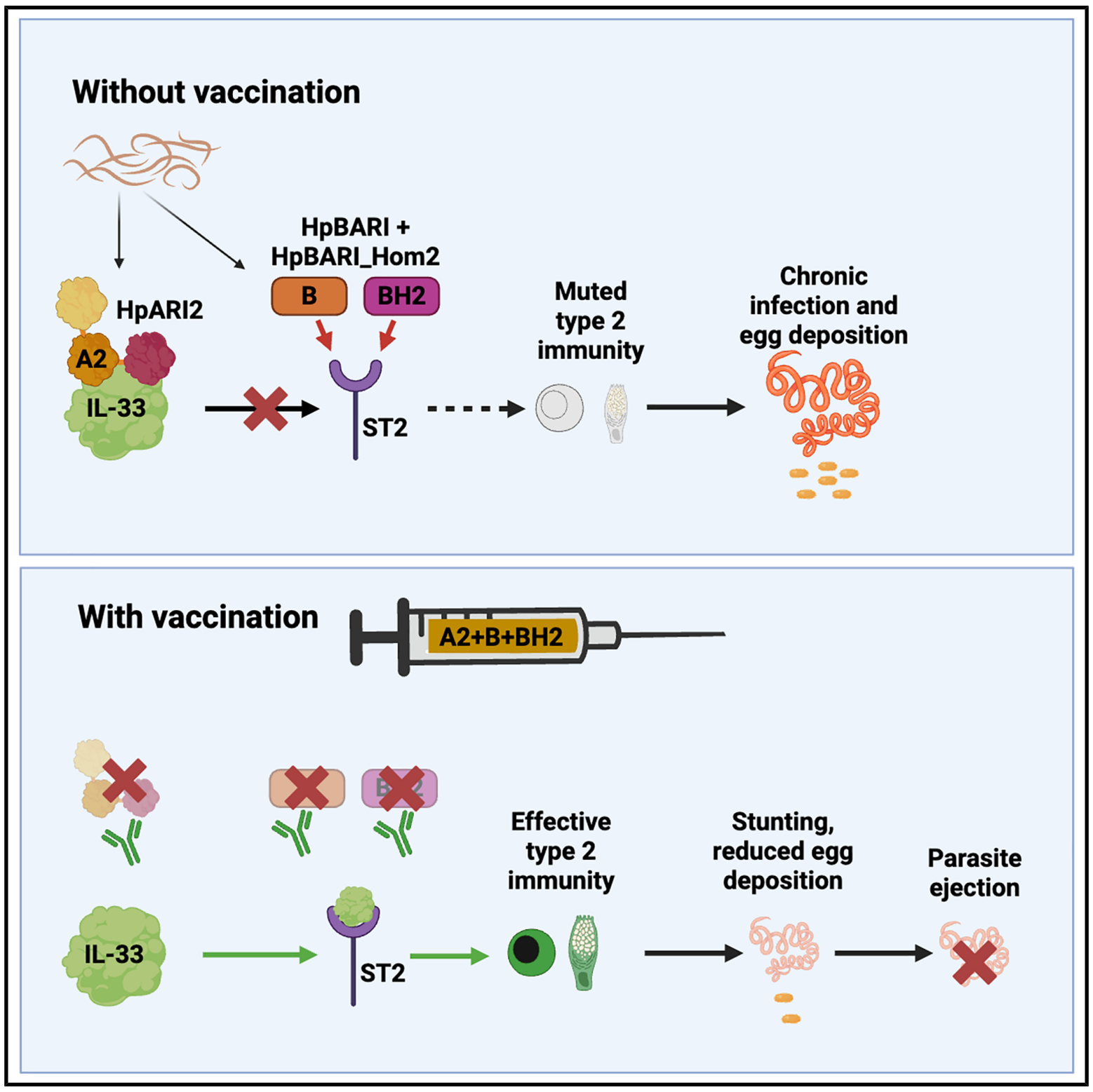

Highlights

- Vaccination with *H. polygyrus bakeri* immunomodulatory proteins allows parasite ejection
- Vaccination releases type 2 immune responses (especially ILC2s) from suppression
- Vaccination raises blocking antibody responses against HpARI2, HpBARI, and HpBARI_Hom2

Authors

Danielle J. Smyth, Suzanne H. Hodge, Nicole W.P. Ong, ..., Marta Campillo Poveda, Rick M. Maizels, Henry J. McSorley

Correspondence

hmcsorley001@dundee.ac.uk

In brief

The helminth immunomodulators from the HpARI and HpBARI families can block IL-33 responses. Smyth and Hodge et al. show that vaccination with these immunomodulatory proteins can raise blocking antibody responses against them, allowing the development of effective anti-parasite type 2 immunity and ejection of the parasite.

Smyth et al., 2025, Cell Reports *44*, 115721
May 27, 2025 © 2025 The Author(s). Published by Elsevier Inc.
https://doi.org/10.1016/j.celrep.2025.115721

Cell Reports

CellPress
OPEN ACCESS

Article

Vaccination against helminth IL-33 modulators permits immune-mediated parasite ejection

Danielle J. Smyth,[1,3] Suzanne H. Hodge,[1,3] Nicole W.P. Ong,[1] Josh Richards,[1] Florent Colomb,[1] Samuele Di Carmine,[1] Vivien Shek,[1] Tania Frangova,[1] Marta Campillo Poveda,[2] Rick M. Maizels,[2] and Henry J. McSorley[1,4,*]
[1]Division of Cell Signalling and Immunology, School of Life Sciences, University of Dundee, Dow Street, Dundee DD1 5EH, UK
[2]Centre for Parasitology, School of Infection and Immunity, University of Glasgow, Glasgow G12 8TA, UK
[3]These authors contributed equally
[4]Lead contact
*Correspondence: hmcsorley001@dundee.ac.uk
https://doi.org/10.1016/j.celrep.2025.115721

SUMMARY

The murine intestinal nematode *Heligmosomoides polygyrus bakeri* (*Hpb*) modulates the host immune response via the *Hpb* alarmin release inhibitor (HpARI) family (HpARI1/2/3), which acts on interleukin (IL)-33, and the *Hpb* binds alarmin receptor and inhibits (HpBARI) family (HpBARI and HpBARI_Hom2), which acts on the IL-33 receptor ST2. Here, we find that this immunomodulation is evident only in the first week of infection and affects local and distal tissues. Vaccination with HpARI or HpBARI proteins raises antibody responses that block their immunomodulatory activities: HpARI2 vaccination results in significantly increased type 2 innate lymphoid cells (ILC2s), T helper (Th)2, and serum IL-4 and IL-5 responses, while HpBARI + HpBARI_Hom2 vaccination reverses infection-mediated ST2 suppression and increases Th2 immunity. A cocktail of HpARI2 + HpBARI + HpBARI_Hom2 gives robust protection against infection, associated with stunting of adult parasites, reduced egg burden, increased type 2 immune responses, and intestinal goblet cell expansion. Therefore, vaccination with immunomodulatory proteins can protect the host against infection and can be used as a tool for blocking the effects of specific parasite-derived proteins.

INTRODUCTION

Intestinal nematode infections affect over 1 billion people worldwide. These parasites can cause significant morbidity (especially in children), including growth stunting, poor school performance, anemia, and malnutrition.[1] Veterinary parasites of livestock are also of large economic importance, and it is estimated that, in Europe, €1.8 billion per year is lost to intestinal nematode infections.[2] Although anthelmintic agents are available for these pathogens, drug resistance is becoming common in livestock parasites, with evidence of development in human-infective nematodes.[3] Furthermore, drug treatments must be given regularly in endemic areas, with rapid reinfection after drug clearance. Therefore, an effective vaccine against helminth infection would be highly advantageous. Currently, there are no vaccines available for any human-infective nematodes, and only very limited vaccines are available against nematodes of veterinary importance.[4] Animal models of helminth infection such as *Heligmosomoides polygyrus bakeri* (*Hpb*) are useful in investigation of anti-parasite immunity and the development of effective anti-parasite vaccines.[5,6] *Hpb* is a widely used model parasite: it is an intestinal nematode of mice and forms chronic infections associated with immunomodulation of bystander responses.[7]

Recently, multiple immunomodulatory proteins have been identified in *Hpb* secretions. They contain multiple unique immunomodulatory protein families, including the *Hpb* alarmin release inhibitors (HpARIs), the *Hpb* binds alarmin receptor and inhibits (HpBARIs), and the *Hpb* transforming growth factor β (TGF-β) mimics, as well as many secreted protein families shared with other parasitic nematodes, such as the apyrases, the DNases, and the venom allergen-like family.[8,9] Of note, both the HpARIs and the HpBARIs act on the same immunological pathway—the interleukin (IL)-33 response. HpARI1 and HpARI2 act directly on IL-33, binding to the cytokine, preventing interaction with its receptor.[10–12] HpARI1 and HpARI2 also bind to DNA and the extracellular matrix, which extends their half-life *in vivo* and allows them to tether IL-33, preventing its release.[10,13] HpARI3 also binds to IL-33 but stabilizes the cytokine, amplifying its effects *in vivo*.[14] The HpBARI family, conversely, act on the IL-33 receptor, ST2. Both HpBARI and HpBARI_Hom2 bind with high affinity to the IL-33 receptor, preventing its interaction with IL-33. We previously showed that HpBARI binding to ST2 was sufficient to abrogate the detection of ST2 on the cell surface using commercially available antibodies; thus, the disappearance of the ST2 signal by flow cytometry can be used as a proxy for the HpBARI family's effects.[15] Our previous work showed that both HpARI2 and HpBARI were capable of suppressing responses in *Alternaria*-induced, IL-33-dependent models of asthma[10,15]; however, due to a lack of transgenesis methodologies in *Hpb*,[16] we could not define a role for these proteins during infection.

This is an open access article under the CC BY license (http://creativecommons.org/licenses/by/4.0/).

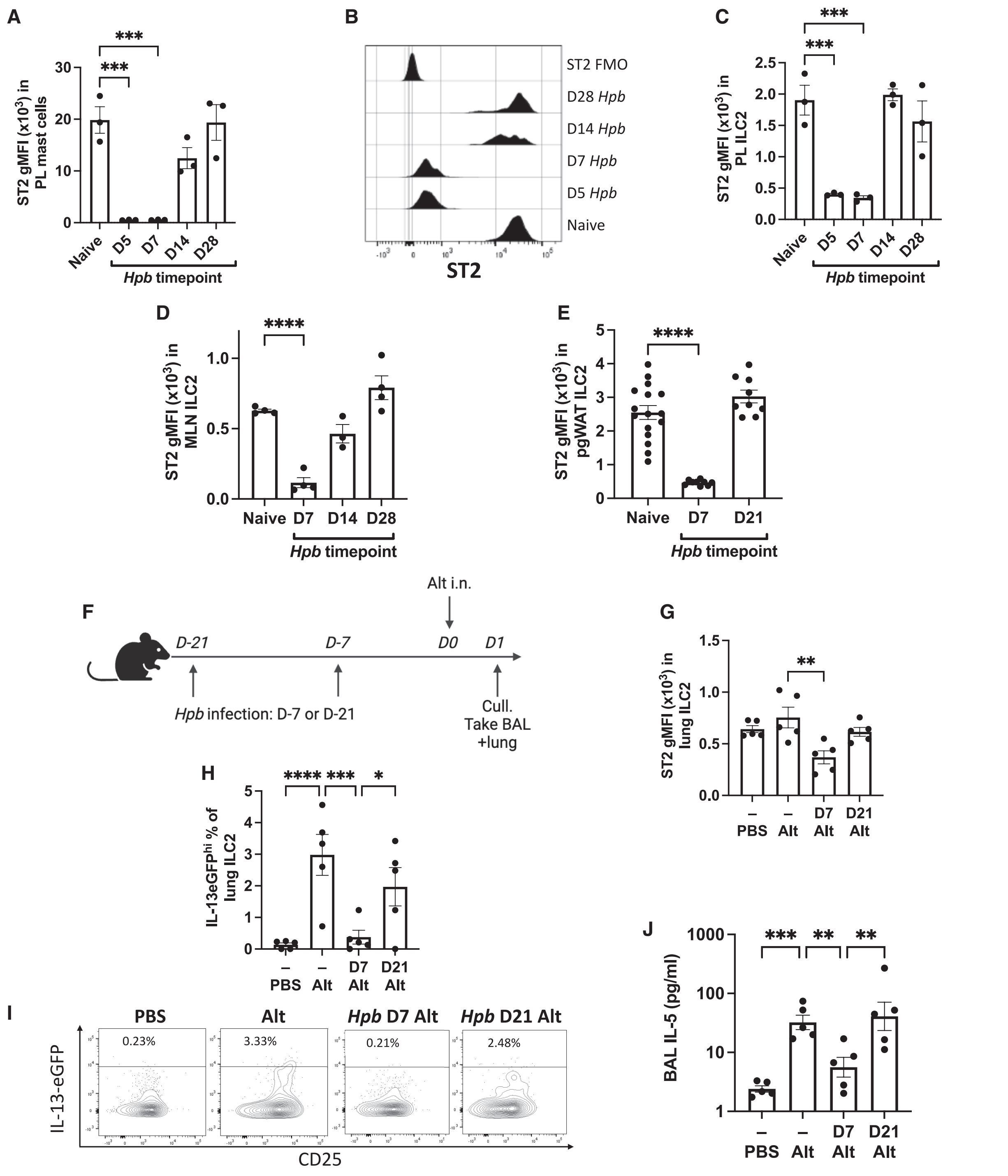

Figure 1. Systemic suppression of IL-33 responses during first week of *Hpb* infection

Mice were infected with *Hpb* and culled at day 5, 7, 14, 21, and 28 of infection as indicated. ST2 geometric mean fluorescence intensity (gMFI) on peritoneal lavage (PL) $CD45^+Lin^-ckit^+Fc\varepsilon R1$ mast cells (A), with representative ST2 histograms on gated mast cells (B). ST2 gMFI on PL $CD45^+Lin^-KLRG1^+$ ILC2s (C), on mesenteric lymph node (MLN) $CD45^+Lin^-CD127^+CD90^+RORgt^-GATA3^+$ ILC2s (D) and on perigonadal white adipose tissue (pgWAT) $CD45^+Lin^-CD25^+KLRG1^+$ ILC2s (E). Experimental protocol for (G)–(J): IL-$13^{+/eGFP}$ mice were infected with *Hpb*, then either 7 days or 21 days later, 50 μg *Alternaria* (Alt) allergen was administered intranasally (i.n.), and mice were sacrificed 1 day later (F). ST2 gMFI on lung $CD45^+Lin^-CD90.2^+CD4^-Fc\varepsilon R1^-$ ILC2s (G), IL-13eGFP^{hi} percentage of lung ILC2s (H), with representative IL-13eGFP versus CD25 bivariate plots of gated ILC2s (I). IL-5 concentration in bronchoalveolar lavage (BAL) (J).

(legend continued on next page)

IL-33 is a pleiotropic cytokine, and depending on the context, IL-33 release can lead to allergy, anti-helminth responses, beiging of adipose tissue, anti-viral responses, or immunosuppression.[17] The IL-33 receptor consists of ST2 and IL1RAcP and is expressed on mast cells, type 2 innate lymphoid cells (ILC2s), T helper (Th)2 cells, regulatory T cells (Tregs), activated Th1, $CD8^+$ T cells, and natural killer (NK) cells.[18] IL-33 receptor signaling on mast cells results in neutrophil recruitment,[19] while signaling on ILC2s and Th2 cells results in the release of IL-5 and eosinophil recruitment.[20] Some populations of Tregs (especially in the adipose tissue and colon) activate and expand in response to IL-33.[21] On Th1, $CD8^+$ T cells, and NK cells, IL-12 treatment upregulates the IL-33 receptor, and subsequent IL-33 signaling is a potent signal for interferon (IFN)-γ release and is important for resistance to viruses.[22] Therefore, the site and context of release is critical to the response to the cytokine.

In this study, we demonstrate that members of both the HpARI and HpBARI immunomodulatory protein families provide protection against infection in a vaccination regime. We furthermore show that vaccination releases the immune system from immunomodulation, through raising blocking antibody responses. These findings provide a mechanistic underpinning and proof of principle that immunomodulatory proteins are good candidates for vaccines against parasitic helminths.

RESULTS

Hpb infection suppresses IL-33-dependent responses during the first week of infection

We previously showed that during *Hpb* infection, the action of the HpBARI family resulted in the suppression of ST2 detection *in vivo* at day 7 of infection.[15] To investigate how sustained and localized this ST2 suppression is, we carried out a time course of *Hpb* infection, using flow cytometry to assess surface ST2 on ILC2s and mast cells (constitutively ST2-positive cells) from the peritoneal cavity, mesenteric lymph node (MLN), perigonadal white adipose tissue, or lung. We found that the profound suppression of ST2 detection was evident on both mast cells and ILC2s from all tissue sites assessed; however, it was restricted to the first week of infection only (Figures 1A–1E). This indicates that the effects of the HpBARIs are systemic, but limited to the early phase of infection when *Hpb* resides within the tissue of the duodenal wall, and that they disappear when *Hpb* emerges into the lumen of the intestine, around day 10.[23]

To determine if this systemic abrogation of ST2 detection was associated with functional suppression of IL-33 responses, we measured the IL-33-dependent response 24 h after intranasal *Alternaria* allergen administration. *Alternaria* allergen administration to the lung causes an acute and potent release of IL-33 due to protease-mediated damage and necrosis of epithelial cells, resulting in the release of ATP and IL-33. IL-5 and IL-13 production in response to *Alternaria* allergen are highly dependent on IL-33 signaling on lung ILC2s.[24–26] In these experiments, we used IL-$13^{+/eGFP}$ mice[20] to allow identification of ILC2 IL-13 production. *Alternaria* was administered to naive IL-$13^{+/eGFP}$ mice or mice infected with *Hpb* 7 days or 21 days prior (Figure 1F). Similar to results seen in other tissue sites, we found that ST2 detection on lung ILC2s was suppressed 7 days after *Hpb* infection and returned to baseline levels by day 21 of infection (Figure 1G). *Alternaria* allergen administration induced strong ILC2 IL-13 production in the lungs of uninfected mice, as well as IL-5 release to the bronchoalveolar lavage (Figures 1H–1J). Soluble IL-4 and IL-13 levels in the bronchoalveolar lavage were below levels of detection. These responses were suppressed at day 7 of *Hpb* infection but returned to similar levels as the positive control by day 21 of *Hpb* infection. However, the eosinophil response in the bronchoalveolar lavage and lung tissue was not suppressed by *Hpb* infection, and in fact, at day 21 of infection, eosinophilia was increased compared to uninfected controls (Figure S5), possibly due to systemic eosinophilia seen during *Hpb* infection.[27] Together, these data indicate that suppression of IL-33-dependent ILC2 responses is limited to the early phase of *Hpb* infection (when *Hpb* expression of HpARI and HpBARI families is highest[14,28]) and rapidly returns to baseline once the parasite enters the lumen of the intestine.

HpARI2 (but not HpARI1 or HpARI3) vaccination abrogates immune suppression

To determine whether this suppression of IL-33 in the first week of *Hpb* infection was dependent on the effects of the HpARI and HpBARI families, we used a vaccination approach to raise a blocking antibody response against each immunomodulatory protein, focusing first on the HpARI family. In non-vaccinated mice, no antibody responses could be detected against any HpARI protein at day 7 of infection, and responses only became evident against specific proteins after day 14 of infection (Figure 2A). No detectable antibody response was raised against HpARI3 at any time point of *Hpb* infection, which may reflect its low expression level.[14] HpARI1, HpARI2, or HpARI3 were administered in a vaccination regime with an alum adjuvant, followed by *Hpb* infection (Figure 2B). Serum antibody responses were assessed at day 7 of infection, when it was again seen that there was a negligible response against HpARI proteins in PBS-vaccinated controls. By contrast, HpARI-vaccinated mice raised a large antibody response against the vaccinated targets. Significant levels of cross-reactivity were seen between HpARIs; however, antibody titers were in each case highest to the vaccinated HpARI family member (Figure 2C). Of note, HpARI vaccination produced antibody titers at least 10-fold higher than that seen at any time point in natural infection (Figure 2A). This antibody response could block the effects of the respective HpARIs in an *in vitro* IL-33 response assay (described in the study by Colomb et al.[14]; Figure S6), with anti-HpARI1 or HpARI2 serum

All data are representative of at least 2 repeat experiments, using mice 8–15 weeks old. In (A)–(D), $n = 3$ per time point, male C57BL/6J mice. In (E), $n = 9$–16 per group, both male and female C57BL/6J mice. In (G)–(J), $n = 5$ per group, male and female IL-13eGFP^{hi} mice. Data are represented as mean ± SEM. Data were analyzed by one-way ANOVA followed by multiple comparison test: Dunnett's when comparing to a single control group (A, C, D, and E), or Šídák's when comparing between multiple groups (G, H, and J). Unless otherwise stated, differences are not significant. *$p < 0.05$, **$p < 0.01$, ***$p < 0.001$, ****$p < 0.0001$, ns = $p > 0.05$.

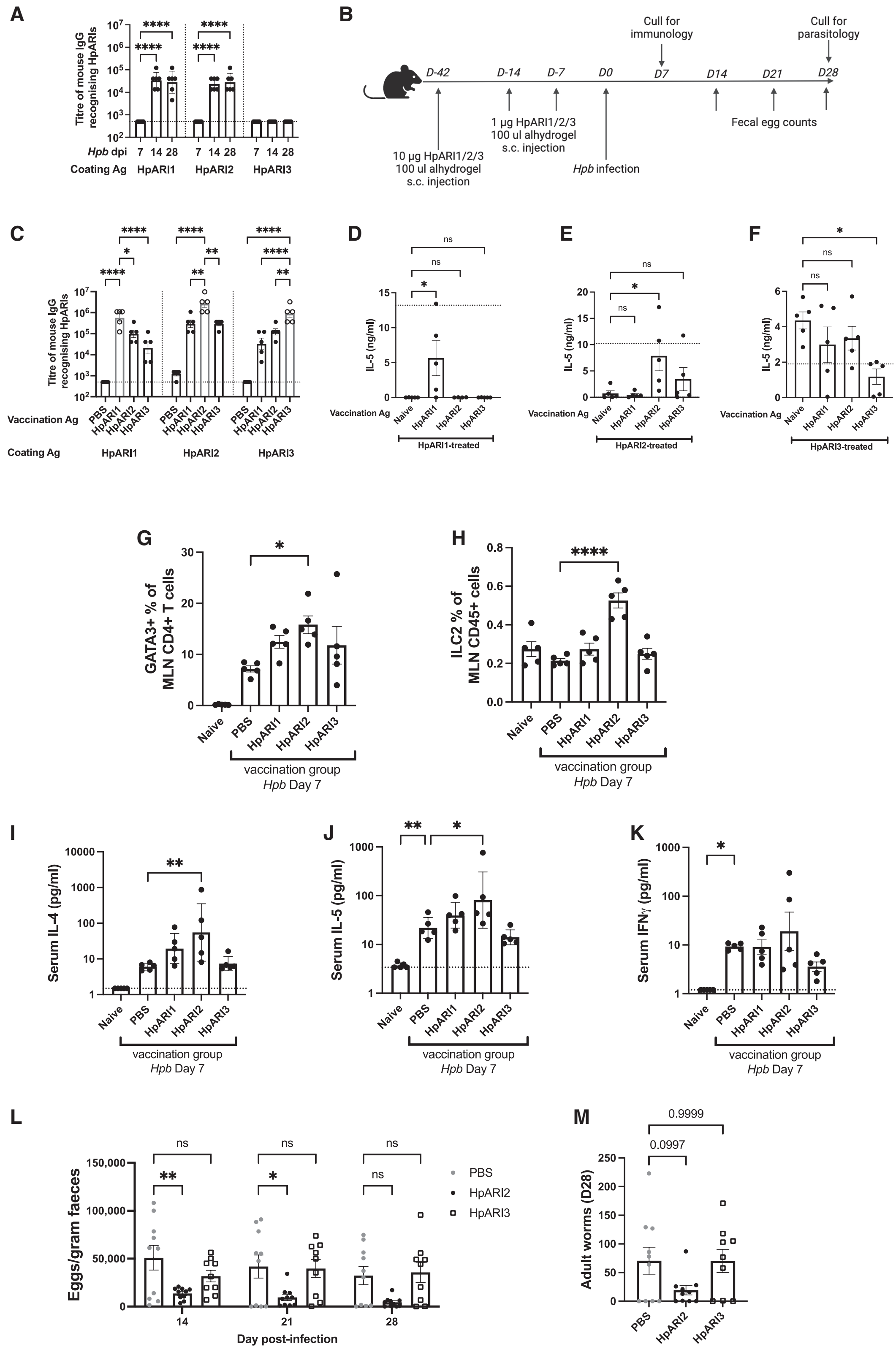

(legend on next page)

preventing the IL-33-blocking effect of HpARI1 and HpARI2, respectively, while anti-HpARI3 serum reduced the known IL-33-amplifying effect of HpARI3 (Figures 2D–2F). *In vivo*, HpARI2 vaccination increased Th2 (Figure 2G) and ILC2 responses (Figure 2H) in the MLN at day 7 of *Hpb* infection, compared to PBS-vaccinated infected controls. Serum IL-4 and IL-5 levels were also increased with HpARI2 vaccination (Figures 2I and 2J), while IFN-γ was unchanged (Figure 2K). Serum IL-13 levels were measured but fell below the limit of detection in all experiments. By each of these measures, HpARI1 had a similar but smaller effect than HpARI2, and trends for increases with HpARI1 did not reach statistical significance in these experiments.

With the significant increase in type 2 immune responses on infection with HpARI2 vaccination, we further investigated the effect of vaccination of HpARI2 on parasite burden. In these experiments, we used HpARI3 as a control vaccine antigen, as it is a closely related protein to HpARI2; however, vaccination with HpARI3 showed no effects on type 2 immunity. HpARI2 vaccination resulted in the reduction in fecal egg counts from day 14 onward (Figure 2L), with a trend for reduction in adult worm burden (Figure 2M). HpARI3 vaccination, by contrast, had no effect on parasite burden.

Together, these data indicate that HpARI2 vaccination results in a blocking antibody response against HpARI2, releasing the immune response from suppression and allowing effective ejection of the parasite.

HpBARI cocktail vaccination abrogates ST2 suppression

The same vaccination approach was taken to assess the role of the HpBARI family. Similar to the response seen against the HpARI family, in natural infection, we found no detectable antibody response against the HpBARIs at day 7 of infection (Figure 3A). While a significant antibody response was raised against HpBARI_Hom2 at later time points of infection, no response could be detected against HpBARI, which, like HpARI3, may reflect HpBARI lower expression level compared to HpBARI_Hom2.[28] Vaccination with HpBARI and/or HpBARI_Hom2 produced high-titer antibody responses against the relevant HpBARI, with some cross-reactivity between the two related proteins (Figure 3B).

To test the effects of blocking the HpBARIs, we first assessed the effect of vaccination on the abrogation of ST2 detection seen previously at day 7 of *Hpb* infection (as in Figures 1A–1C). HpBARI family vaccination was compared to HpARI2 vaccination, as HpARI2 does not act on ST2 directly.

We found that vaccination with either HpBARI or HpBARI_Hom2 alone was not capable of fully reversing the ST2 suppression seen at day 7 of infection, but vaccination with both proteins in combination resulted in ST2 detection on peritoneal cavity mast cells and ILC2s returning to (or exceeding) levels seen in uninfected mice (Figures 3C–3E). HpARI2 vaccination, by contrast, did not significantly affect ST2 staining on these populations. Although the effect on peritoneal mast cell ST2 was particularly pronounced, this is likely to be due to the extremely high level of ST2 expression of these cells: mast cell responses appeared unaffected with similar levels of mast cell protease (MCPT)4 (released on connective tissue mast cell degranulation) found in the serum and peritoneal lavage of vaccinated mice (Figure S7), while MCPT1 (marker of mucosal mast cell degranulation) could not be detected in any group at this time point. Therefore, these data indicate that both HpBARI and HpBARI_Hom2 are required to fully suppress ST2 at day 7 of *Hpb* infection.

HpBARI cocktail vaccination releases type 2 immunity from suppression and allows parasite ejection

We investigated the effects of HpBARI family vaccination in the MLN, finding that similar to the peritoneal cavity, suppression of ST2 detection on ILC2s or Th2 was abrogated by combined HpBARI + HpBARI_Hom2 vaccination (Figures 4A and 4B). Surprisingly, in the MLN (but not in the peritoneal cavity), abrogation of ST2 suppression was achieved even with sole HpBARI_Hom2 vaccination (but not with sole HpBARI vaccination) indicating a site-specific difference in effects of these two immunomodulatory homologs. The combined HpBARI + HpBARI_Hom2 vaccination also increased type 2 immune responses, with increased proportions of Th2 cells (Figure 4C), but unlike HpARI2 vaccination (as in Figure 2E), there was no significant increase in ILC2 responses (Figure 4D). As with HpARI2 vaccination, increased type 2 immunity with HpBARI + HpBARI_Hom2 vaccination resulted in increased serum IL-4 and IL-5 (Figures 4E and 4F), but in contrast to HpARI2 vaccination, it also increased serum IFN-γ (Figure 4G). There was a smaller, but still significant, effect of HpBARI_Hom2 vaccination alone, with no effect of HpBARI vaccination on these Th2 parameters. Altogether, these results

Figure 2. Vaccination with HpARI2 releases type 2 response from suppression and protects against *Hpb* infection

Mice were infected with *Hpb*, and HpARI1, HpARI2, and HpARI3-specific serum IgG titers were measured at 7, 14, or 28 days post infection (dpi) (A). Experimental protocol for vaccination experiments using HpARI1, HpARI2, or HpARI3 followed by *Hpb* infection (B). HpARI1, HpARI2, and HpARI3-specific serum IgG titers after vaccination, at day 7 of *Hpb* infection (C). Serum from vaccinated, day 7 *Hpb*-infected mice was tested for blocking activity in an *in vitro* IL-33 response assay, to abrogate the effects of HpARI1 (D), HpARI2 (E), or HpARI3 (F) on IL-33-dependent IL-5 secretion. Mesenteric lymph nodes were taken from vaccinated mice at day 7 of *Hpb* infection, to measure GATA3^{+}Foxp3^{-} percentage of MLN CD4^{+}Lin^{+}CD45^{+} T cells (G) and GATA3^{+}Lin^{-} ILC2 percentage of MLN CD45^{+} cells (H). Serum was taken from vaccinated mice at day 7 of *Hpb* infection, to measure concentrations of IL-4 (I), IL-5 (J), and IFN-γ (K). At days 14, 21, and 28 of *Hpb* infection after vaccination, *Hpb* eggs were counted in fecal samples (L). At day 28 of infection after vaccination, mice were culled, and adult worm burdens were assessed (M).

Dotted line on *x* axis indicates limit of detection in (A), (C), (I), (J), and (K). In (D), (E), and (F), dotted line indicates level of IL-5 response in the absence of the relevant HpARI protein. Data are represented as mean ± SEM. Data are representative of at least 2 repeat experiments, apart from HpARI1 group, which is from a single experiment. (A), (C), (D), (E), (F), (G), (H), (I), (J), (K), and (M) were analyzed by one-way ANOVA, and (L) was analyzed by two-way ANOVA, all with Dunnett's multiple comparisons test. In (A), all groups were compared to day 7 of infection; in (C), all groups were compared to relevant vaccinated group (indicated in gray); in (D)–(J), all groups were compared to PBS-vaccinated *Hpb* D7 control. (A, C, D, E, F, G, and H) $n = 5$ per group, (I and J) $n = 10$ per group. Female C57BL/6J mice aged 6–12 weeks used in all experiments. Unless otherwise stated, differences are not significant. *$p < 0.05$, **$p < 0.01$, ***$p < 0.001$, ****$p < 0.0001$, ns = $p > 0.05$.

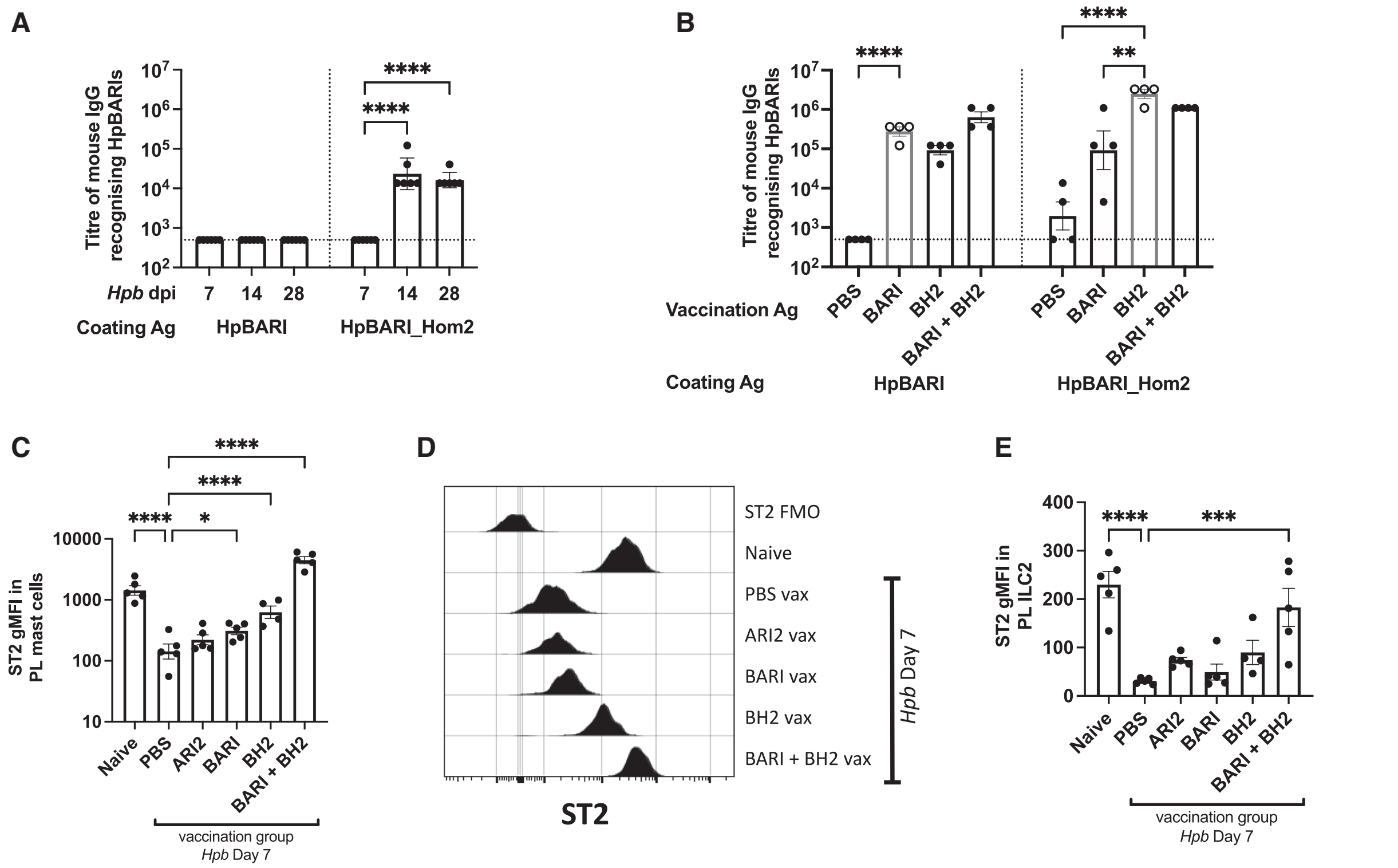

Figure 3. HpBARI family vaccination abrogates ST2 suppression

Mice were infected with *Hpb* and serum taken 7, 14, and 28 days post infection (dpi) to measure HpBARI or HpBARI_Hom2-specific serum IgG titers (A). Mice were vaccinated with HpARI2 (ARI2), HpBARI (BARI), or HpBARI_Hom2 (BH2) as indicated, and then infected with *Hpb* and culled at day 7 of infection. HpBARI (BARI) and HpBARI_Hom2 (BH2)-specific serum IgG responses (B). ST2 gMFI on peritoneal lavage (PL) $CD45^+Lin^-ckit^+(Fc\varepsilon R1^+$ or $IgE^+)$ mast cells (C) with representative histograms of ST2 on gated PL mast cells (D). ST2 gMFI on PL $CD45^+Lin^-KLRG1^+$ ILC2s (E).

Dotted line on *x* axis indicates limit of detection, where relevant. Data are represented as mean ± SEM. Analyzed by one-way ANOVA with Dunnett's multiple comparisons test, comparing all groups to PBS-vaccinated *Hpb* D7 control. Unless otherwise indicated, differences are not significant. Data are representative of 2 repeat experiments. Female C57BL/6J mice aged 6–12 weeks used in all experiments. In (A), $n = 6$ per group; in (B), $n = 4$ per group; in (C)–(E), $n = 4$–5 per group. $^*p < 0.05$, $^{**}p < 0.01$, $^{***}p < 0.001$, $^{****}p < 0.0001$, ns = $p > 0.05$.

indicate that to fully reverse the ST2 suppression seen in the early phases of *Hpb* infection, a cocktail of HpBARI + HpBARI_Hom2 is most effective.

HpBARI + HpBARI_Hom2 vaccination, alone or in combination with HpARI2, stunts adult worms and reduces parasite burden

As HpBARI + HpBARI_Hom2 (B + BH2) vaccination abrogated ST2 suppression, we hypothesized that this would also result in parasite ejection. Similarly, as HpARI2 vaccination was permissive for effective type 2 immunity, we also tested a cocktail of HpARI2 + HpBARI + HpBARI_Hom2 (A2 + B + BH2). Mice were vaccinated with these cocktails of proteins, while another *Hpb* parasite-secreted protein expressed in the same expression system (HPOL_0001072601) was used as a control protein. HpBARI + HpBARI_Hom2 vaccination resulted in significantly reduced egg burdens from day 14 onward and a trend for reduced adult burdens at day 28 (Figures 5A and 5B). The cocktail of HpARI2 + HpBARI + HpBARI_Hom2 was particularly effective, giving almost sterilizing immunity by day 28. To assess whether the vaccine-induced immunity against the parasite was mediated by early ejection, we repeated HpARI2 + HpBARI + HpBARI_Hom2 cocktail vaccination and assessed adult worm burden at day 14 (Figure 5C). Surprisingly, despite showing robustly decreased fecal egg counts at this time point (Figures 5A and 5D), no reduction in adult worm burden could be seen. When fecundity (i.e., egg counts divided by adult worm counts) was calculated (Figure 5E), vaccination could be seen to reduce the egg output of adult parasites. Adult parasites from these mice were smaller and appeared to have reduced internal egg deposits (Figures 5F and 5G). Therefore, vaccination, and the concurrent release of type 2 immunity, may result in stunting of developing parasites, reduced egg deposition, and ejection of the majority of adult parasites between days 14 and 28.

HpARI2 + HpBARI + HpBARI_Hom2 vaccination results in increased type 2 immunity in the draining lymph node and intestine

We assessed whether the cocktail vaccination was still capable of raising robust antibody responses against each immunomodulatory protein and found very similar IgG titers as with each single vaccine immunization (Figures S8, 2C, and 3B). We then assessed ST2 detection on MLN ILC2s and found that the

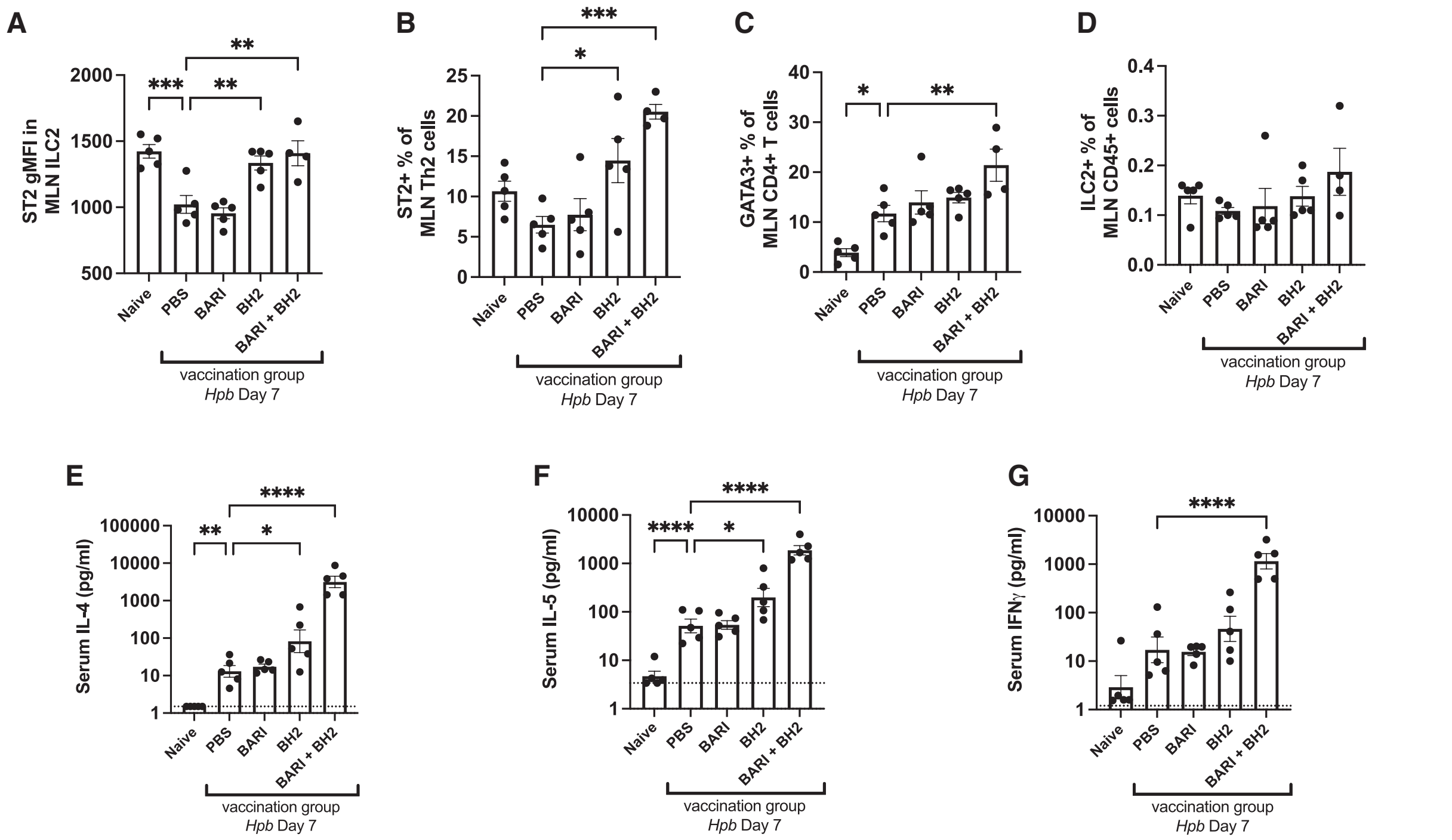

Figure 4. HpBARI family vaccination releases type 2 response from suppression

Mice were vaccinated with HpBARI (BARI) and/or HpBARI_Hom2 (BH2) and then infected with *Hpb*. At day 7 of infection, ST2 gMFI on MLN GATA3^{+}CD127^{+}Lin^{-}CD45^{+} ILC2s (A), ST2^{+} percentage of MLN GATA3^{+}CD4^{+}Lin^{+}CD45^{+} Th2 cells (B), GATA3^{+} percentage of MLN CD4^{+}Lin^{+}CD45^{+} Th cells (C), and ILC2 percentage of MLN CD45^{+} cells (D) were assessed by flow cytometry. Serum IL-4 (E), IL-5 (F), and IFN-γ (G) were measured by LEGENDplex cytokine bead array.

Dotted line on *y* axis indicates limit of detection, where relevant. Data are represented as mean ± SEM. All data are representative of 2 repeat experiments. (A–G) analyzed by one-way ANOVA with Dunnett's multiple comparisons test, In (A)–(G), comparisons are made to PBS-vaccinated *Hpb* D7 control. In all panels, $n = 4$–5 per group. Female C57BL/6J mice aged 6–12 weeks used in all experiments. Unless otherwise stated, differences are not significant. *$p < 0.05$, **$p < 0.01$, ***$p < 0.001$, ****$p < 0.0001$, ns = $p > 0.05$.

cocktail vaccine was capable of reversing ST2 suppression at day 7, while at day 14, ILC2 ST2 expression was increased compared to naive controls (Figure 6A). This was associated with increased ILC2 responses, as seen previously with HpARI2 vaccination at day 7 of infection (Figure 2H), and although this did not reach significance in this experiment at day 7, at day 14 of infection, a highly significant expansion of ILC2s was evident (Figure 6B). This ILC2 expansion was accompanied by a more modest increase in Th2 cells (Figure 6C). IL-33 signaling has been implicated in Treg expansion[21]; however, little change in Foxp3+ Tregs could be detected with vaccination, with a modest increase only at day 14 of infection (Figure 6D). The increase in early type 2 immune responses was communicated to the site of infection, as increased numbers of periodic acid-Schiff-positive goblet cells could be seen in vaccinated mice at day 7, a time point where no significant goblet cell induction could be seen in PBS-vaccinated controls (Figures 6E and 6F). Finally, when cytokine responses were assessed in the MLN at day 14 of infection, we found that while PBS-vaccinated control mice did not show any detectable IL-5/IL-13 ILC2 response at this time point, ILC2 cytokine production was robustly induced in cocktail-vaccinated day 14 *Hpb*-infected mice (Figures 6G–6I). Furthermore, while an MLN Th2 IL-5/IL-13 response could be detected in day 14 *Hpb*-infected PBS-vaccinated mice, this was much increased on cocktail vaccination (Figures 6H–6I). These data support a pathway where HpARI2, HpBARI, and HpBARI_Hom2 block IL-33-dependent responses early in *Hpb* infection, and abrogation of this immunomodulation using a vaccination approach allows effective mucosal type 2 immunity (especially ILC2 responses), which leads to parasite stunting and ejection.

DISCUSSION

Hpb produces a range of immunomodulatory proteins, which have been proposed to allow persistence of the parasite *in vivo*, preventing the development of effective immunity. Here, we show that vaccination with members of these immunomodulatory families was able to both induce clearance of the parasite and abrogate the effects of these immunomodulatory proteins. This work therefore provides a proof of principle that these secreted proteins inhibit parasite ejection in a non-redundant manner, that immunomodulatory proteins have potential as vaccine candidates, and that vaccination can allow us to investigate the roles of these proteins *in vivo*.

An intractable problem in studies of parasitic helminths is the lack of suitable transgenesis systems. Here, we show that vaccination allowed us to block specific parasite proteins *in vivo*, to

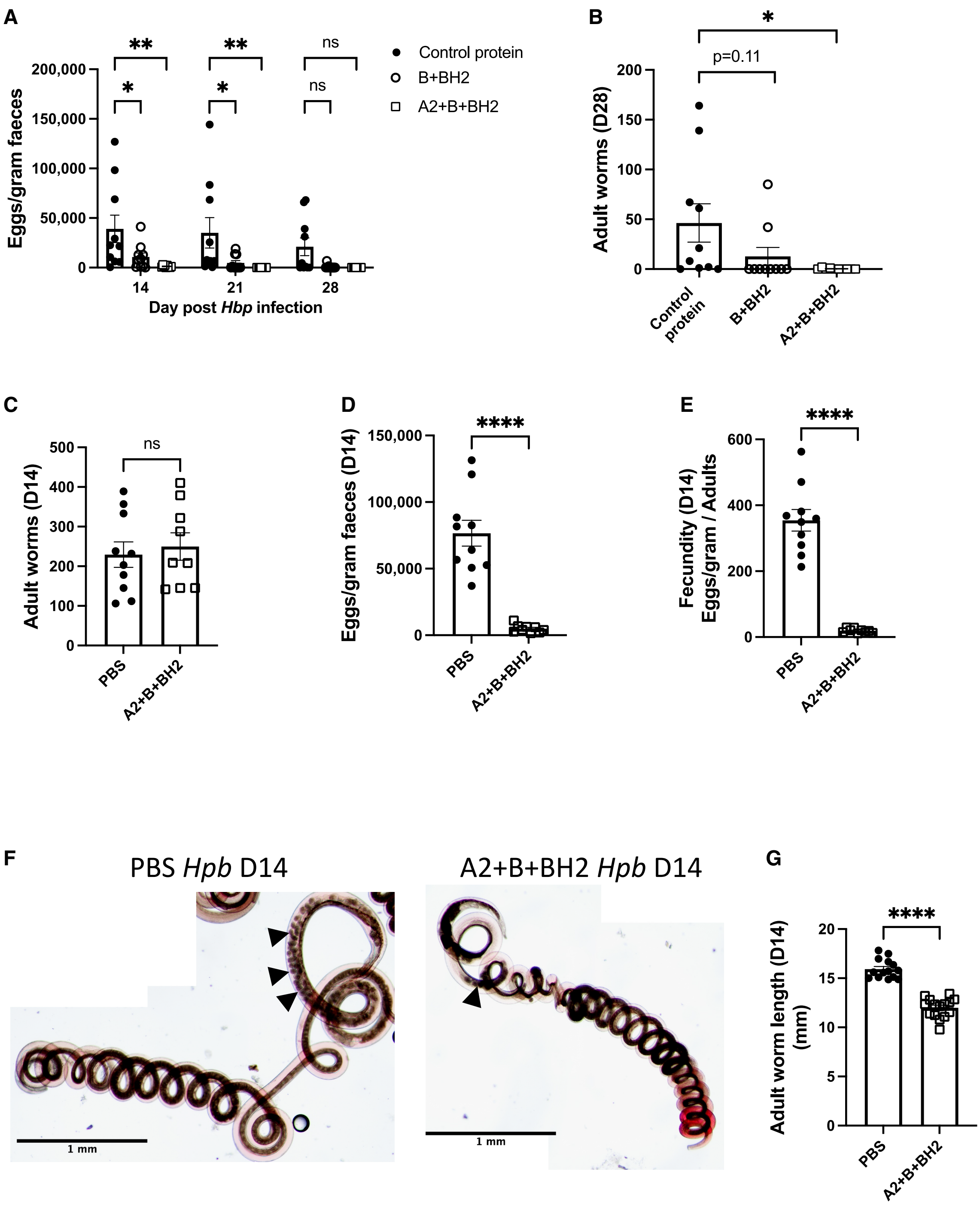

Figure 5. Cocktail vaccinations result in damage and ejection of the parasite

Mice were vaccinated with HPOL_0001072601 control protein, HpBARI + HpBARI_Hom2 (B + BH2), or HpARI2 + HpBARI + HpBARI_Hom2 (A2 + B + BH2): at day 14, 21, and 28 of *Hpb* infection, fecal egg counts were taken (A), and mice were culled at day 28 of infection to adult worm burdens (B). In an independent experiment, mice were vaccinated with PBS or A2 + B + BH2 and culled at day 14 for adult worm burdens (C), fecal egg counts (D), and calculation of fecundity (egg count per mouse divided by adult burden, E). Representative photomicrographs of female adult worms from day 14 of infection (F) (arrows indicate eggs inside female parasites) and measurements of female parasite length (G). Scale bars indicate 1 mm.

(legend continued on next page)

assess their importance in parasite persistence. To our surprise, we found that members of the HpARI and HpBARI families, which had similar effects *in vitro*, had quite different effects when blocked *in vivo*. For instance, HpARI1 and HpARI2 similarly suppress IL-33 *in vitro*,[14] but only HpARI2 vaccination had a significant effect on the type 2 immune response during *Hpb* infection. Conversely, despite our recent findings that HpARI3 can stabilize IL-33 *in vivo*,[14] vaccination had no effects on type 2 immunity during *Hpb* infection. In contrast, while HpBARI and HpBARI_Hom2 have very similar effects on the suppression of ST2 *in vitro*,[15] only vaccination with a combination of both proteins (and not each protein alone) fully abrogated ST2 suppression and allowed active type 2 immunity to eject the parasite. Therefore, our vaccination experiments indicate that the HpBARI family members act in a coordinated manner to block ST2, while vaccination with the HpARI or HpBARI families shows that these sets of proteins have non-redundant roles in infection.

As both the HpARI and HpBARI families act on the IL-33-ST2 pathway, we might expect that abrogating their effects through vaccination would result in identical outcomes. Indeed, vaccinating with either HpARI2 or HpBARI + HpBARI_Hom2 resulted in increased Th2 responses, increased IL-4 and IL-5 levels in the serum, and increased resistance to the parasite, indicating that the effects of both of these sets of proteins are required for suppression of the immune response and parasite persistence. However, some qualitative differences were identified between HpARI2 and HpBARI + HpBARI_Hom2 vaccinations: HpARI2 vaccination resulted in much increased ILC2 proportions in the draining lymph node (on which HpBARI + HpBARI_Hom2 vaccination had no effect), while HpBARI + HpBARI_Hom2 vaccination resulted in increased serum IFN-γ, which was not seen in HpARI2 vaccination. The reasons for the disparity between HpARI2 and HpBARI + HpBARI_Hom2 vaccination are unclear, but could be due to non-classical IL-33 signaling. It was recently shown that oxidized human IL-33 was found to activate an (ST2-independent) RAGE-EGFR pathway.[29] Our previous publications indicate that while HpARI2 binds only to reduced, and not oxidized, IL-33, its binding may prevent the oxidation of the cytokine[10,12]; therefore, HpARI2 may block both the ST2-dependent and RAGE-EGFR-dependent pathways, similarly to the anti-IL-33 antibody tozorakimab, which potently inhibits IL-33-mediated peripheral blood mononuclear cell IFN-γ responses.[30] Meanwhile, the HpBARI family's effects appear constrained to ST2[15] and would not affect oxidized IL-33 signaling. Another potential explanation for the difference in effects of HpARI versus HpBARI vaccination could be their site of activity: we recently showed that HpARI2 binds to the extracellular matrix, which we propose acts in retention and extension of effective half-life at the site of deposition.[13] Conversely, the data presented here show that the HpBARIs' effects are evident throughout the mouse but rapidly fade after the parasite exits the intestinal wall. Therefore, timing and cellular context may explain the differences in the effects of HpARI versus HpBARI blockade.

As the effects of HpARI2 or HpBARI + HpBARI_Hom2 vaccination appeared to be non-redundant, we then assessed the effects of an HpARI2 + HpBARI + HpBARI_Hom2 triple cocktail vaccine. We found that this regime showed the most profound immunity to *Hpb* infection, while strongly increasing type 2 responses (especially ILC2 responses) in the draining lymph node and in the small intestine. To our surprise, the reduction in egg burden from the earliest time points measured (i.e., day 14 post infection) did not correlate with reduced adult burden: at day 14, similar numbers of adult parasites could be found in the lumen of the gut, but those from vaccinated mice appeared smaller and contained fewer eggs. Therefore, we propose that vaccination with these immunomodulatory proteins releases the immune response from immunosuppression at early time points, resulting in damage and stunting (but not killing) of adult parasites, reducing egg burdens, and allowing more rapid ejection of parasites between the second and fourth weeks of infection. These data are in contrast to those seen using vaccination with total excretory/secretory products of *Hpb* (HES), which show a reduction in adult worm burden at day 14,[5,31] indicating that the effects of HES vaccination may at least partially depend on responses raised to other antigens than the HpARI and HpBARI families.

Suppression of the IL-33 pathway by *Hpb* appears constrained to the early phases of infection, with suppression of IL-33-dependent responses in the *Alternaria* model and abrogation of ST2 detection only evident during the first week of infection. The transcription patterns of the HpARI and HpBARI family members correlate with this—while HpARI1 and HpARI3 remain relatively constant throughout the *Hpb* life cycle, transcription of HpARI2 peaks in the first week of infection and is rapidly reduced thereafter.[14] Similarly, while HpBARI transcription remains stable throughout infection, HpBARI_Hom2 also peaks in the first week of infection.[28] Therefore, this first week of infection appears to be a critical window of immunomodulatory activity. During the first week of infection, *Hpb* resides within the wall of the duodenum, emerging into the lumen as adults at around day 7–10. Therefore, during this critical early time period, *Hpb* can release immunomodulatory factors directly into the tissue. The first week of *Hpb* infection is also associated with a weak type 1 response at the site of infection, which only switches to a type 2 response once the parasite enters the lumen.[32] The action of the HpARI and HpBARI families may therefore inhibit the IL-33-mediated development of Th2 immunity until the parasite exits the tissue.

Although the HpARI and HpBARI families are clearly important to the immunomodulatory activity of *Hpb*, they are not the only immunomodulatory family secreted by the parasite. Many helminths, including *Hpb*, secrete active apyrases, and a cocktail vaccine consisting of 5 recombinant apyrases could provide

Data are represented as mean ± SEM. (A) was analyzed by two-way ANOVA with Dunnett's multiple comparisons test, while (B) was analyzed by one-way ANOVA with Dunnett's multiple comparisons test, in each case comparing to control protein vaccination. (C), (D), (E), and (G) were analyzed by unpaired t test. Female C57BL/6J mice aged 6–12 weeks used in all experiments, with $n = 10$ per group in (A)–(E). For (F) and (G), adult parasites were taken at random from 4 mice per group, and 3–4 parasites measured per mouse, to give a total $n = 13–14$ per group. Unless otherwise stated, differences are not significant. *$p < 0.05$, **$p < 0.01$, ***$p < 0.001$, ****$p < 0.0001$, ns = $p > 0.05$.

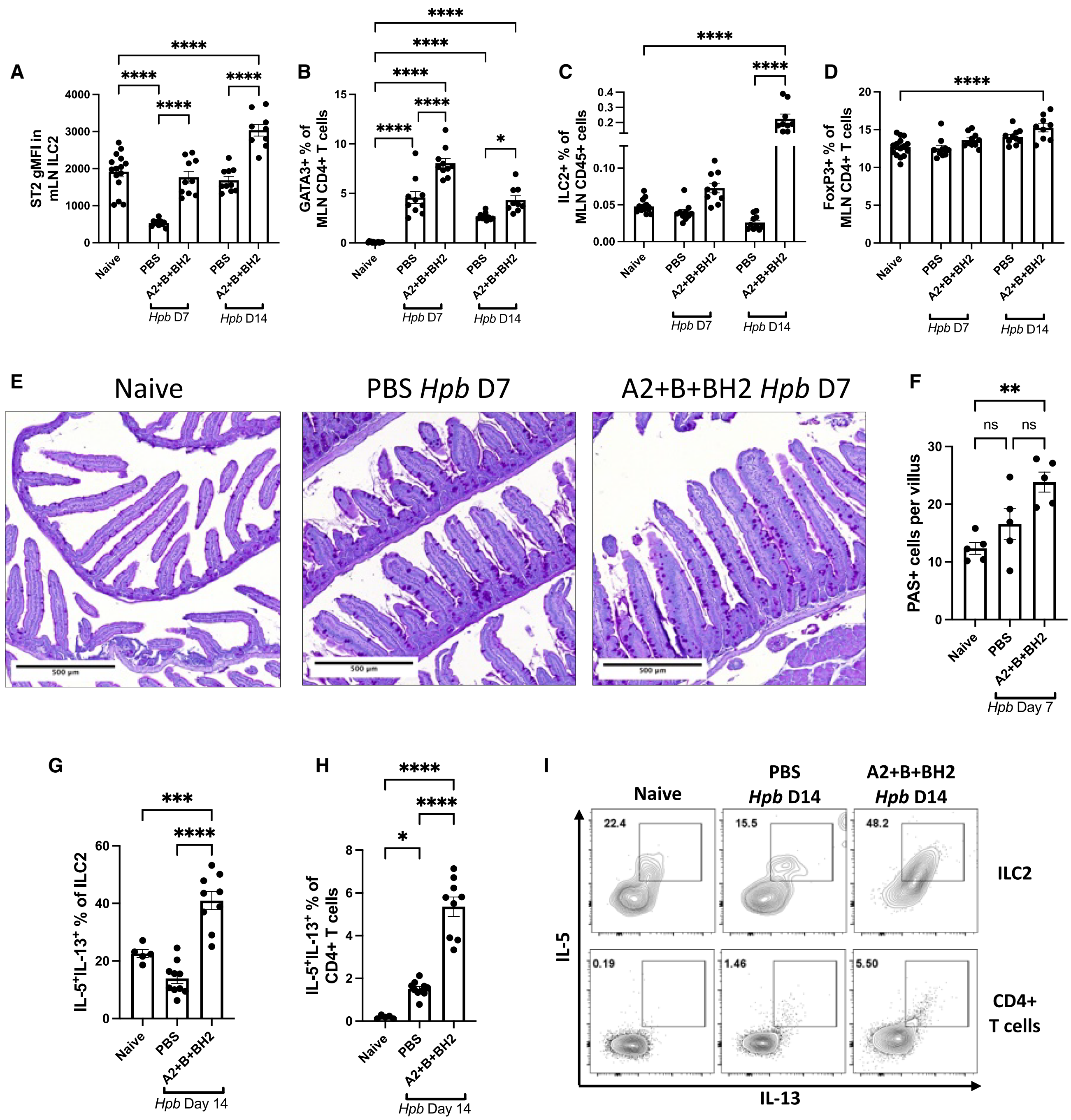

Figure 6. HpARI2 + HpBARI + HpBARI_Hom2 cocktail vaccination allows effective type 2 immunity

Mice were infected with *Hpb* after vaccination with HpARI2 + HpBARI + HpBARI_Hom2 (A2 + B + BH2) or PBS-vaccinated controls. At day 7 or 14 after infection, MLN cells were taken and stained for flow cytometry analysis of ST2 gMFI on ILC2s (A), GATA3+ percentage of $CD4^+$ T cells (B), ILC2+ percentage of live $CD45^+$ cells (C), and Foxp3+ percentage of $CD4^+$ T cells (D). At day 7 of infection, formalin-fixed small intestinal gut rolls were stained by periodic acid-Schiff (PAS), representative images shown in (E) (scale bars indicate 500 µm), and PAS+ cells per villus (average of 2 independent blinded scores) shown in (F). At day 14 of infection, MLN cells were stimulated with 1x eBioscience Cell Stimulation cocktail plus protein transport inhibitor and stained for intracellular cytokines by flow cytometry: IL-5+IL-13+ percentage of ILC2s shown in (G), IL-5+IL-13+ percentage of $CD4^+$ T cells shown in (H), and representative bivariate plots shown in (I). Data are represented as mean ± SEM. Data were analyzed by one-way ANOVA followed by multiple comparison test: Šídák's when comparing naive controls to all other groups, and PBS-vaccinated to A2 + B + BH2-vaccinated groups at each time point (A, B, C, and D), or Tukey's when comparing all groups against one

(legend continued on next page)

CellPress
OPEN ACCESS

partial protection from *Hpb* infection.[33] Likewise, *Hpb* secretes the TGF-β mimics (TGM) family, which ligates the mammalian TGF-β receptor.[34,35] Although this family has been well characterized *in vitro*, its effects during infection are less well understood—vaccinating mice with TGM family members could allow dissection of their functions.

Our work indicates that parasite-secreted immunomodulators can be used as vaccine antigens in helminth infection. Previous data in other parasite models support this: vaccination with the *Trichuris muris* IL-13-inhibitory protein p43 provides protection against infection.[36] In parasites of livestock, putative immunomodulatory proteins have been tested as vaccines and have also shown protection against infection.[37] While the data presented here represent an important proof of principle that immunomodulatory proteins can be used as effective vaccines in parasitic infections, they cannot be directly translated to human infections, as no homologs of HpARI or HpBARI have been identified in any human-infective parasites. As there are currently no vaccines available for any human parasitic helminths, further investigation of parasite-specific immunomodulatory secreted products could lead to the identification of effective vaccine candidates.

In summary, we provide a proof of principle that vaccination with immunomodulatory parasite proteins can result in blocking antibody responses against these proteins, releasing the immune response from suppression, and allowing productive immunity to the parasite. This research highlights the importance of the identification of helminth immunomodulatory proteins for development as vaccine candidates and provides a tool for investigation of immunomodulatory function during infection.

Limitations of the study

This study is limited by the specificity of the responses studied to the helminth parasite *Hpb*—the immunomodulators released by this mouse-infective parasite are the best understood of any intestinal helminth; however, no homologs of HpARI or HpBARI have yet been identified in any related parasite of humans or any other mammal. Therefore, these findings cannot be directly translated to similar parasites but instead provide a proof of principle that vaccination with immunomodulatory proteins provides protection from parasitic infections. Furthermore, although this study shows abrogation of the HpARI and HpBARI family's activities via blocking antibody responses, we cannot discount the role of T cell priming in vaccination, driving a larger type 2 response against vaccine antigens. On this point, it is especially interesting that ILC2 responses (which are not antigen specific) are particularly boosted by HpARI2 vaccination. A better approach to investigate the effects of HpARI and HpBARI would be to create HpARI/HpBARI-deficient parasites; however, no stable transgenic *Hpb* parasites have yet been generated. Availability of transgenesis in parasitic helminth biology would greatly accelerate research into host-parasite interactions.

RESOURCE AVAILABILITY

Lead contact

Requests for further information and resources should be directed to and will be fulfilled by the lead contact, Dr. Henry J. McSorley (hmcsorley001@dundee.ac.uk).

Materials availability

All unique reagents generated in this study are available from the lead contact with a completed materials transfer agreement.

Data and code availability

- All data reported in this paper will be shared by the lead contact upon request.
- This paper does not report original code.
- Any additional information required to reanalyze the data reported in this paper is available from the lead contact upon request.

ACKNOWLEDGMENTS

This work was funded by a Wellcome Investigator award (221914/Z/20/Z) to H.J.M. We thank Prof. Georgia Perona-Wright and Prof. Constance Finney for feedback and advice on the manuscript. Some figures were created in BioRender. McSorley, H. (2025) https://BioRender.com/e16b389. For the purpose of open access, the author has applied a Creative Commons Attribution (CC BY) license to any Author Accepted Manuscript version arising from this submission.

AUTHOR CONTRIBUTIONS

D.J.S., S.H.H., and H.J.M. conceived and planned the study and wrote the manuscript. D.J.S., S.H.H., N.W.P.O., J.R., V.S., and H.J.M. conducted mouse experiments. T.F. conducted protein production and quality control. F.C. carried out *in vitro* IL-33 assays. D.J.S. and T.F. maintained parasite life cycle and parasitology measurements. D.J.S., S.D.C., M.C.P., and R.M.M. planned and carried out histological staining and analysis. All authors designed the experiments, analyzed the data, and read and commented on the manuscript.

DECLARATION OF INTERESTS

The authors declare no competing interests.

STAR★METHODS

Detailed methods are provided in the online version of this paper and include the following:

- KEY RESOURCES TABLE
- EXPERIMENTAL MODEL AND STUDY PARTICIPANT DETAILS
  - Animals
  - Vaccination and Hpb infection
- METHOD DETAILS
  - Protein production
  - Tissue preparation for flow cytometry
  - Flow cytometry
  - Serum antibody and cytokine measurements
  - IL-33 response assay
  - Histology
- QUANTIFICATION AND STATISTICAL ANALYSIS

another (F, G, and H). Female C57BL/6J mice aged 6–12 weeks used in all experiments. Data in (A)–(D) are pooled from 2 repeat experiments, for a total of $n = 9$–10 per group. Data in (E) and (F) are from a single experiment with $n = 5$ per group. Data in (G)–(I) are pooled from 2 repeat experiments, for a total group size of $n = 5$ for naive, $n = 10$ for PBS vaccination, and $n = 9$ for A2 + B + BH2 vaccination. Unless otherwise stated, differences are not significant. *$p < 0.05$, **$p < 0.01$, ***$p < 0.001$, ****$p < 0.0001$, ns = $p > 0.05$.

SUPPLEMENTAL INFORMATION

Supplemental information can be found online at https://doi.org/10.1016/j.celrep.2025.115721.

Received: September 16, 2024
Revised: March 19, 2025
Accepted: April 29, 2025
Published: May 15, 2025

REFERENCES

1. Jourdan, P.M., Lamberton, P.H.L., Fenwick, A., and Addiss, D.G. (2018). Soil-transmitted helminth infections. Lancet *391*, 252–265.
2. Charlier, J., Rinaldi, L., Musella, V., Ploeger, H.W., Chartier, C., Vineer, H. R., Hinney, B., von Samson-Himmelstjerna, G., Băcescu, B., Mickiewicz, M., et al. (2020). Initial assessment of the economic burden of major parasitic helminth infections to the ruminant livestock industry in Europe. Prev. Vet. Med. *182*, 105103.
3. Ng'etich, A.I., Amoah, I.D., Bux, F., and Kumari, S. (2023). Anthelmintic resistance in soil-transmitted helminths: One-Health considerations. Parasitol. Res. *123*, 62.
4. Vercruysse, J., Charlier, J., Van Dijk, J., Morgan, E.R., Geary, T., von Samson-Himmelstjerna, G., and Claerebout, E. (2018). Control of helminth ruminant infections by 2030. Parasitology *145*, 1655–1664.
5. Hewitson, J.P., Filbey, K.J., Esser-von Bieren, J., Camberis, M., Schwartz, C., Murray, J., Reynolds, L.A., Blair, N., Robertson, E., Harcus, Y., et al. (2015). Concerted activity of IgG1 antibodies and IL-4/IL-25-dependent effector cells trap helminth larvae in the tissues following vaccination with defined secreted antigens, providing sterile immunity to challenge infection. PLoS Pathog. *11*, e1004676.
6. Bieren, E.V., Volpe, B., Kulagin, M., Sutherland, D.B., Guiet, R., Seitz, A., Marsland, B.J., Verbeek, J.S., and Harris, N.L. (2015). Antibody-mediated trapping of helminth larvae requires CD11b and Fcgamma receptor I. J. Immunol. *194*, 1154–1163.
7. Maizels, R.M., Hewitson, J.P., Murray, J., Harcus, Y.M., Dayer, B., Filbey, K.J., Grainger, J.R., McSorley, H.J., Reynolds, L.A., and Smith, K.A. (2012). Immune modulation and modulators in *Heligmosomoides polygyrus* infection. Exp. Parasitol. *132*, 76–89.
8. Maizels, R.M., Smits, H.H., and McSorley, H.J. (2018). Modulation of Host Immunity by Helminths: The Expanding Repertoire of Parasite Effector Molecules. Immunity *49*, 801–818.
9. Lothstein, K.E., and Gause, W.C. (2021). Mining Helminths for Novel Therapeutics. Trends Mol. Med. *27*, 345–364.
10. Osbourn, M., Soares, D.C., Vacca, F., Cohen, E.S., Scott, I.C., Gregory, W. F., Smyth, D.J., Toivakka, M., Kemter, A.M., le Bihan, T., et al. (2017). HpARI Protein Secreted by a Helminth Parasite Suppresses Interleukin-33. Immunity *47*, 739–751.e5.
11. Jamwal, A., Colomb, F., McSorley, H.J., and Higgins, M.K. (2024). Structural basis for IL-33 recognition and its antagonism by the helminth effector protein HpARI2. Nat. Commun. *15*, 5226.
12. Chauche, C., Vacca, F., Chia, S.L., Richards, J., Gregory, W.F., Ogunkanbi, A., Wear, M., and McSorley, H.J. (2020). A Truncated Form of HpARI Stabilizes IL-33, Amplifying Responses to the Cytokine. Front. Immunol. *11*, 1363.
13. Colomb, F., Jamwal, A., Ogunkanbi, A., Frangova, T., Savage, A.R., Kelly, S., Wright, G.J., Higgins, M.K., and McSorley, H.J. (2024). Heparan sulphate binding controls in vivo half-life of the HpARI protein family. Elife *13*, RP99000.
14. Colomb, F., Ogunkanbi, A., Jamwal, A., Dong, B., Maizels, R.M., Finney, C.A.M., Wasmuth, J.D., Higgins, M.K., and McSorley, H.J. (2024). IL-33-binding HpARI family homologues with divergent effects in suppressing or enhancing type 2 immune responses. Infect. Immun. *92*, e0039523.
15. Vacca, F., Chauché, C., Jamwal, A., Hinchy, E.C., Heieis, G., Webster, H., Ogunkanbi, A., Sekne, Z., Gregory, W.F., Wear, M., et al. (2020). A helminth-derived suppressor of ST2 blocks allergic responses. Elife *9*, e54017.
16. Quinzo, M.J., Perteguer, M.J., Brindley, P.J., Loukas, A., and Sotillo, J. (2022). Transgenesis in parasitic helminths: a brief history and prospects for the future. Parasit. Vectors *15*, 110.
17. Cayrol, C., and Girard, J.P. (2018). Interleukin-33 (IL-33): A nuclear cytokine from the IL-1 family. Immunol. Rev. *281*, 154–168.
18. McSorley, H.J., and Smyth, D.J. (2021). IL-33: A central cytokine in helminth infections. Semin. Immunol. *53*, 101532.
19. Enoksson, M., Möller-Westerberg, C., Wicher, G., Fallon, P.G., Forsberg-Nilsson, K., Lunderius-Andersson, C., and Nilsson, G. (2013). Intraperitoneal influx of neutrophils in response to IL-33 is mast cell-dependent. Blood *121*, 530–536.
20. Neill, D.R., Wong, S.H., Bellosi, A., Flynn, R.J., Daly, M., Langford, T.K.A., Bucks, C., Kane, C.M., Fallon, P.G., Pannell, R., et al. (2010). Nuocytes represent a new innate effector leukocyte that mediates type-2 immunity. Nature *464*, 1367–1370.
21. Schiering, C., Krausgruber, T., Chomka, A., Fröhlich, A., Adelmann, K., Wohlfert, E.A., Pott, J., Griseri, T., Bollrath, J., Hegazy, A.N., et al. (2014). The alarmin IL-33 promotes regulatory T-cell function in the intestine. Nature *513*, 564–568.
22. Yang, Q., Li, G., Zhu, Y., Liu, L., Chen, E., Turnquist, H., Zhang, X., Finn, O. J., Chen, X., and Lu, B. (2011). IL-33 synergizes with TCR and IL-12 signaling to promote the effector function of CD8+ T cells. Eur. J. Immunol. *41*, 3351–3360.
23. Johnston, C.J.C., Robertson, E., Harcus, Y., Grainger, J.R., Coakley, G., Smyth, D.J., McSorley, H.J., and Maizels, R. (2015). Cultivation of *Heligmosomoides polygyrus*: an immunomodulatory nematode parasite and its secreted products. J. Vis. Exp. *98*, e52412.
24. Bartemes, K.R., Iijima, K., Kobayashi, T., Kephart, G.M., McKenzie, A.N., and Kita, H. (2012). IL-33-responsive lineage- CD25+ CD44(hi) lymphoid cells mediate innate type 2 immunity and allergic inflammation in the lungs. J. Immunol. *188*, 1503–1513.
25. Kouzaki, H., Iijima, K., Kobayashi, T., O'Grady, S.M., and Kita, H. (2011). The danger signal, extracellular ATP, is a sensor for an airborne allergen and triggers IL-33 release and innate Th2-type responses. J. Immunol. *186*, 4375–4387.
26. McSorley, H.J., Blair, N.F., Smith, K.A., McKenzie, A.N.J., and Maizels, R. M. (2014). Blockade of IL-33 release and suppression of type 2 innate lymphoid cell responses by helminth secreted products in airway allergy. Mucosal Immunol. *7*, 1068–1078.
27. Strandmark, J., Steinfelder, S., Berek, C., Kühl, A.A., Rausch, S., and Hartmann, S. (2017). Eosinophils are required to suppress Th2 responses in Peyer's patches during intestinal infection by nematodes. Mucosal Immunol. *10*, 661–672.
28. Pollo, S.M.J., Leon-Coria, A., Liu, H., Cruces-Gonzalez, D., Finney, C.A. M., and Wasmuth, J.D. (2023). Transcriptional patterns of sexual dimorphism and in host developmental programs in the model parasitic nematode *Heligmosomoides bakeri*. Parasit. Vectors *16*, 171.
29. Strickson, S., Houslay, K.F., Negri, V.A., Ohne, Y., Ottosson, T., Dodd, R. B., Huntington, C.C., Baker, T., Li, J., Stephenson, K.E., et al. (2023). Oxidised IL-33 drives COPD epithelial pathogenesis via ST2-independent RAGE/EGFR signalling complex. Eur. Respir. J. *62*, 2202210.
30. England, E., Rees, D.G., Scott, I.C., Carmen, S., Chan, D.T.Y., Chaillan Huntington, C.E., Houslay, K.F., Erngren, T., Penney, M., Majithiya, J.B., et al. (2023). Tozorakimab (MEDI3506): an anti-IL-33 antibody that inhibits IL-33 signalling via ST2 and RAGE/EGFR to reduce inflammation and epithelial dysfunction. Sci. Rep. *13*, 9825.
31. Hewitson, J.P., Ivens, A.C., Harcus, Y., Filbey, K.J., McSorley, H.J., Murray, J., Bridgett, S., Ashford, D., Dowle, A.A., and Maizels, R.M. (2013). Secretion of protective antigens by tissue-stage nematode larvae revealed

by proteomic analysis and vaccination-induced sterile immunity. PLoS Pathog. *9*, e1003492.

32. Gentile, M.E., Li, Y., Robertson, A., Shah, K., Fontes, G., Kaufmann, E., Polese, B., Khan, N., Parisien, M., Munter, H.M., et al. (2020). NK cell recruitment limits tissue damage during an enteric helminth infection. Mucosal Immunol. *13*, 357–370.
33. Berkachy, R., Smyth, D.J., Schnoeller, C., Harcus, Y., Maizels, R.M., Selkirk, M.E., and Gounaris, K. (2021). Characterisation of the secreted apyrase family of *Heligmosomoides polygyrus*. Int. J. Parasitol. *51*, 39–48.
34. Johnston, C.J.C., Smyth, D.J., Kodali, R.B., White, M.P.J., Harcus, Y., Filbey, K.J., Hewitson, J.P., Hinck, C.S., Ivens, A., Kemter, A.M., et al. (2017). A structurally distinct TGF-beta mimic from an intestinal helminth parasite potently induces regulatory T cells. Nat. Commun. *8*, 1741.
35. Smyth, D.J., Harcus, Y., White, M.P.J., Gregory, W.F., Nahler, J., Stephens, I., Toke-Bjolgerud, E., Hewitson, J.P., Ivens, A., McSorley, H.J., and Maizels, R.M. (2018). TGF-beta mimic proteins form an extended gene family in the murine parasite *Heligmosomoides polygyrus*. Int. J. Parasitol. *48*, 379–385.
36. Bancroft, A.J., Levy, C.W., Jowitt, T.A., Hayes, K.S., Thompson, S., Mckenzie, E.A., Ball, M.D., Dubaissi, E., France, A.P., Bellina, B., et al. (2019). The major secreted protein of the whipworm parasite tethers to matrix and inhibits interleukin-13 function. Nat. Commun. *10*, 2344.
37. Nisbet, A.J., McNeilly, T.N., Wildblood, L.A., Morrison, A.A., Bartley, D.J., Bartley, Y., Longhi, C., McKendrick, I.J., Palarea-Albaladejo, J., and Matthews, J.B. (2013). Successful immunization against a parasitic nematode by vaccination with recombinant proteins. Vaccine *31*, 4017–4023.
38. Preibisch, S., Saalfeld, S., and Tomancak, P. (2009). Globally optimal stitching of tiled 3D microscopic image acquisitions. Bioinformatics *25*, 1463–1465.
39. Howe, K.L., Bolt, B.J., Shafie, M., Kersey, P., and Berriman, M. (2017). WormBase ParaSite - a comprehensive resource for helminth genomics. Mol. Biochem. Parasitol. *215*, 2–10.

STAR★METHODS

KEY RESOURCES TABLE

REAGENT or RESOURCE	SOURCE	IDENTIFIER
Antibodies		
Fixable Aqua Dead Cell Stain Kit	Invitrogen	Cat# L34966
Zombie UV Fixable Viability Kit	BioLegend	Cat# 423108
Anti-mouse CD16/32 (Clone: 93, 1:50)	BioLegend	Cat# 101302; RRID: AB_312801
CD127-FITC (clone: A7R34, 1:200)	BioLegend	Cat# 135007; RRID: AB_1937231
CD44-PerCP (clone: IM7, 1:200)	BioLegend	Cat# 103031; RRID: AB_2076206
CD90.2-AF700 (clone: 30-H12, 1:200)	BioLegend	Cat# 105320; RRID: AB_493725
CD45-APCCy7 (clone: I3/2.3, 1:200)	BioLegend	Cat# 147718; RRID: AB_2888795
CD45-AF700 (clone: 30-F11, 1:200)	BioLegend	Cat# 103128; RRID: AB_493715
CD3-Pacific Blue (clone: 17A2, 1:200)	BioLegend	Cat# 100214; RRID: AB_493645
CD3-Biotin (clone: 145-2C11, 1:200)	BioLegend	Cat# 100304; RRID: AB_312669
CD3-FITC (clone: 145-2C11, 1:200)	BioLegend	Cat# 100306; RRID: AB_312671
CD5-Pacific Blue (clone: 53–7.3, 1:200)	BioLegend	Cat# 100642; RRID: AB_2813916
CD5-FITC (clone: 53–7.3, 1:200)	Invitrogen	Cat# 11-0051-85; RRID: AB_464909
NK1-1-Pacific Blue (clone: PK136, 1:200)	BioLegend	Cat# 108722; RRID: AB_2132712
B220-Pacific Blue or -Biotin (clone: RA3-6B2, 1:200)	BioLegend	Cat# 103227; RRID: AB_492876; Cat# 103204; RRID: AB_312989
CD11b-Pacific Blue, -FITC or -Biotin (clone: M1/70, 1:200)	BioLegend	Cat# 101224; RRID: AB_755986; Cat# 101206; RRID: AB_312789; Cat# 101203; RRID: AB_312786
CD11c-Pacific Blue or -AF647 (clone: N418, 1:200)	BioLegend	Cat# 117322; RRID: AB_755988; Cat# 117312; RRID: AB_389328
ST2-Biotin (clone: DIH9, 1:100)	BioLegend	Cat# 145308; RRID: AB_2565569
ST2-APC (clone: RMST2-2, 1:100)	Invitrogen	Cat# 17-9335-82; RRID: AB_2573301
CD25-BrilliantViolet 650 (clone: PC61, 1:200)	BioLegend	Cat# 102038; RRID: AB_2563060
CD4-BrilliantViolet 711 (clone: GK1.5, 1:200)	BioLegend	Cat# 100447; RRID: AB_2564586
CD4-PE-Dazzle (clone: RM4-5, 1:200)	BioLegend	Cat# 100565; RRID: AB_2563684
KLRG1-PE-Dazzle or -PerCP (clone: 2F1/KLRG1, 1:200)	BioLegend	Cat# 138424; RRID: AB_2564051; Cat# 138418; RRID: AB_2563015
Gr1-Biotin or -FITC (clone: RB6-8C5, 1:200)	BioLegend	Cat# 108404; RRID: AB_313369; Cat# 108405; RRID: AB_313370
Ter-119-Biotin (clone: TER-119, 1:200)	BioLegend	Cat# 116204; RRID: AB_313705
CD19-FITC (clone: 6D5, 1:200)	BioLegend	Cat# 115506; RRID: AB_313641
FcεRI-PE-Cy7 (clone: MAR-1, 1:200)	BioLegend	Cat# 134318; RRID: AB_10640122
cKit-Pacific Blue or -PerCP (clone: 2B8, 1:200)	BioLegend	Cat# 105820; RRID: AB_493476; Cat# 105823; RRID: AB_2131598
ICOS-PE (clone: C398.4A, 1:200)	BioLegend	Cat# 313508; RRID: AB_416332
IgE-PE (clone: RME-1, 1:200)	BioLegend	Cat# 406907; RRID: AB_493291
IL-5-PE (clone: TRFK5, 1:50)	Biolegend	Cat# 504303; RRID: AB_315327
IL-13-PE-Cy7 (clone: eBio13A, 1:50)	Invitrogen	Cat# 25-7133-82; RRID: AB_2573530
Streptavidin-BrilliantViolet 510 or -PE (1:100)	BioLegend	Cat# 405234; Cat# 405203

(*Continued on next page*)

CellPress
OPEN ACCESS

Continued

REAGENT or RESOURCE	SOURCE	IDENTIFIER
CD49b–FITC (clone: DX5, 1:200)	Invitrogen	Cat# 11-5971-85; RRID: AB_465328
Siglec-F-PE (clone: REA798, 1:200)	Miltenyi Biotec	Cat# 130-112-174; RRID: AB_2653440
GATA3-PE (clone: TWAJ, 1:50)	Invitrogen	Cat# 12-9966-42; RRID: AB_1963600
RORγt-APC (clone: B2D, 1:100)	Invitrogen	Cat# 17-6981-80; RRID: AB_2573253
FoxP3-PE-Cy7 (clone: FJK-16s, 1:100)	Invitrogen	Cat# 25-5773-82; RRID: AB_891552
Horseradish peroxidase-conjugated Goat anti-Mouse IgG (H + L) (1:2000)	BioRad	Cat# 1706516; RRID: AB_2921252
IL-5-PE (clone TRFK5, 1:100)	BioLegend	Cat# 504304; RRID: AB_315328
IL-13-PE-Cy7 (clone eBio13A, 1:100)	Invitrogen	Cat# 25-7133-82; RRID: AB_2573530
**Chemicals, peptides, and recombinant proteins**		
Alhydrogel adjuvant 2%	InvivoGen	Cat# vac-alu-250
Recombinant HpARI1	Colomb F et al.[14]	NA
Recombinant HpARI2	Colomb F et al.[14]	NA
Recombinant HpARI3	Colomb F et al.[14]	NA
Recombinant HpBARI	Vacca F et al.[15]	NA
Recombinant HpBARI_Hom2	Vacca F et al.[15]	NA
Recombinant control protein (HPOL_0001072601)	This Paper	NA
Recombinant Murine IL-2 (carrier free)	BioLegend	Cat# 575402
Recombinant Murine IL-7 (carrier free)	BioLegend	Cat# 577802
**Critical commercial assays**		
Murine uncoated IL-5 ELISA	Invitrogen	Cat# 88-7054-77
LEGENDplex murine Th1/Th2 8 -plex Version 3	BioLegend	Cat# 741054
MCPT4 ELISA kit (Mouse)	Insight Biotechnology	Cat# OKWB00279
HEK-Blue LPS Detection kit 2	InvivoGen	Cat# rep-lps2
**Experimental models: Cell lines**		
Human: CMT64 cell line	ATCC	ECACC 10032301; RRID:CVCL_2406
**Experimental models: Organisms/strains**		
Mouse: C57BL/6J (JAX Mice Strain)	Charles River UK	Strain code 632
Mouse: Heterozygous IL-13$^{+/eGFP}$ (C57BL/6J background)	Neill DR et al.[20]	Prof Andrew McKenzie
Parasite: *Heligmosomoides polygyrus bakeri*	Maizels RM et al.[7]	NA
Allergen: *Alternaria alternata*	Greer	Cat# XPM1D3A25
**Software and algorithms**		
Prism version 10.2.3	Graphpad	www.graphpad.com
FlowJo version 10.9	BD Biosciences	www.flowjo.com
Fiji (ImageJ2 version 2.16.0)	Open Source	
**Other**		
eBioscience FoxP3 fixation/ permeabilization set	Invitrogen	Cat# 11500597
Liberase TM Research Grade	Roche	Cat# 5401119001
Liberase TL Research Grade	Roche	Cat# 05401020001
Deoxyribonuclease I from bovine pancreas	Sigma	Cat# D5025-150KU
eBioscience Cell Stimulation Cocktail (500x)	Invitrogen	Cat# 00-4975-93

EXPERIMENTAL MODEL AND STUDY PARTICIPANT DETAILS

Animals

C57BL/6JCrl mice were purchased from Charles River, UK, while heterozygous IL-13$^{+/eGFP}$ mice on a C57BL/6J background were provided by Prof Andrew McKenzie.[20] Mouse accommodation and procedures were performed under UK Home Office licenses (project license PP9520011) with institutional oversight performed by qualified veterinarians. Experiments were approved by the local Welfare and Ethical use of Animals in Research committee (WEC), and conform to relevant regulatory standards. Mouse sex and age, as well as biological replicates per group in each experiment are stated in figure legends.

Vaccination and Hpb infection

Mice were vaccinated with recombinant HpARI1, HpARI2, HpARI3, HpBARI, HpBARI_Hom2 or control protein (HPOL_0001072601), or PBS controls received PBS in adjuvant only. Mice were subcutaneously injected in the flank with 10 μg of parasite protein in 100 μL Alhydrogel (InvivoGen) alum adjuvant, followed by two boosts of 1 μg of parasite protein in adjuvant 28 and 35 days later. In combination vaccinations (HpBARI + HpBARI_Hom2 or HpARI2 + HpBARI + HpBARI_Hom2), 10 μg (primary) or 1 μg (boost) of each protein were used in combination. At day 42 after the first injection, mice were infected with 200 *Hpb* L3 larvae by oral gavage. Mice were culled 7 days after infection for measurement of immune responses, and day 14 or 28 days after infection for adult worm counts. Fecal pellets were collected at day 14, 21 and 28 of infection for egg counts. Adult worms were counted manually under a dissecting microscope. Eggs per gram of feces counts were carried out by resuspending weighed fecal pellets in 1 mL water, vortexing to create a slurry, then mixing with 1 mL of a saturated salt solution. Egg counts of both chambers of a McMaster slide were taken per sample and averaged for a final count. To measure the *Hpb* female adult lengths, multiple photomicrographs of each worm were taken on a dissecting microscope. Images were stitched together using the pairwise stitching plugin[38] in ImageJ.

In some experiments, mice were infected with *Hpb* without vaccination and culled at various timepoints as indicated in the figures. In other experiments, uninfected or *Hpb*-infected mice were intranasally administered 50 μg Alternaria allergen (Greer, XPM1D3A25) under isoflurane anesthesia, and culled 24 h later.

METHOD DETAILS

Protein production

As described previously, HpARI1, HpARI2, HpARI3 proteins were expressed recombinantly with C-terminal myc and 6His tags,[14] while HpBARI and HpBARI_Hom2 were expressed recombinantly with N-terminal 6His and myc tags.[15] The HPOL_0001072601 (control protein) sequence is available at Wormbase ParaSite[39] and was expressed recombinantly with an N-terminal 6His tag. All sequences were cloned into a pSecTAG2A vector backbone (Thermo Fisher Scientific), and transfected into mammalian Expi293F cells using the Expifectamine transfection kit (Thermo Fisher Scientific). Proteins were purified from cell-free supernatants 7 days after transfection using Ni-NTA chromatography, and subsequently dialyzed into 1 x PBS, filter sterilized and assayed for LPS levels using the HEK-Blue LPS Detection kit 2 (InvivoGen). All proteins used in these experiments contain <0.1 EU/ug LPS.

Tissue preparation for flow cytometry

Bronchoalveolar lavage (BAL): BAL was collected using a 26G needle inserted into the trachea of the mouse and 4 × 0.5 mL washes made using 1 x PBS. Cells were separated from the BAL fluid by centrifuging at 300 g for 5 min at 4°C. Pelleted cells were used for flow cytometry, while supernatants were stored at −70°C for cytokine ELISA.

Peritoneal lavage (PL): Peritoneal cells were collected by 2 × 5 mL lavages with ice-cold 1 x PBS in the peritoneal cavity. Cells were separated from the wash fluid by centrifugation at 400*g* for 5 min.

Perigonadal White Adipose Tissue (pgWAT): Perigonadal adipose tissue pads were dissected, weighed and minced into small pieces using scissors. Tissue was placed into 1 mL digestion mixture comprising of 1 x PBS^{++} (PBS containing magnesium chloride and calcium chloride; Gibco) containing Liberase TM (50 μg/mL; Roche) and DNAse1 (160 U/ml; Sigma) and placed in a shaking incubator for 35 min at 200 rpm, 37°C. Following digestion, 10 mM EDTA (Gibco) was added and cells were incubated without shaking for a further 5 min. Bijous were topped up with ice-cold FACS buffer (1 x PBS, 0.5% BSA [Sigma]) and samples were filtered through a 70 μm filter (Greiner), and centrifuged at 300 g for 15 min at 4°C. After centrifugation, the adipocyte layer was carefully removed, and the remaining stromal vascular fraction (SVF) was collected for flow cytometry staining.

Mesenteric Lymph Node: The mesenteric lymph nodes (MLN) were taken from each mouse and crushed through a 70 μm nylon filter (Greiner) then resuspended in complete RPMI media (RPMI-1640 (Gibco) supplemented with 10% FCS (Invitrogen), 100 U/ml penicillin, 100 μg/mL streptomycin (Gibco) and 2 mM L-glutamine (Gibco)) for counting and flow cytometry staining.

Lung: Either half (complete lobe) or three-quarters (complete lobe plus largest segmented lobe) were taken from each mouse and collected in 900 μL 1x PBS^{++}. Liberase TL (2 U/ml; Roche) and DNAse1 (160 U/ml; Sigma) were added to each collection tube and lungs were thoroughly minced with scissors. The minced lungs were incubated for 35 min, shaking at 200 rpm at 37°C. The digest was then stopped with 5 mL ice-cold complete RPMI (Gibco) and crushed through a 70 μm nylon filter (Greiner).

For all samples, red blood cells were lysed in ACK lysis buffer (Gibco), then resuspended in PBS for counting and flow cytometry staining.

Flow cytometry

Single cell suspensions of cells were prepared as described as above before being resuspended in 1 x PBS containing a LIVE/DEAD fixable dye (Fixable Aqua Dead Cell Stain Kit (1:1,000, Invitrogen); or Zombie UV Fixable Viability Kit (1:200, BioLegend)) for 15 min in the dark at room temperature. Cells were centrifuged (400 g, 5 min, 4°C) and resuspended in FACS buffer (1 x PBS supplemented with 0.5% BSA) containing purified anti mouse CD16/32 (Clone: 93, BioLegend, 1:50) and incubated at 4°C for 20 min in the dark. Cells were then washed with FACS buffer, centrifuged as before and resuspended in FACS buffer containing extracellular antibodies and incubated for 30 min at 4°C in the dark.

Extracellular antibodies used: CD127-FITC (clone: A7R34, 1:200); CD44-PerCP (clone: IM7, 1:200); CD90.2-AF700 (clone: 30-H12, 1:200); CD45-APCCy7 (clone: I3/2.3, 1:200), CD45-AF700 (clone: 30-F11, 1:200); CD3-Pacific Blue (clone: 17A2, 1:200), CD3-Biotin (clone: 145-2C11, 1:200), CD3-FITC (clone: 145-2C11, 1:200); CD5-Pacific Blue (clone: 53–7.3, 1:200); NK1-1-Pacific Blue (clone: PK136, 1:200); B220-Pacific Blue or -Biotin (clone: RA3-6B2, 1:200); CD11b-Pacific Blue, -FITC or -Biotin (clone: M1/70, 1:200); CD11c-Pacific Blue or -AF647 (clone: N418, 1:200); ST2-Biotin (clone: DIH9, 1:100); CD25-BrilliantViolet 650 (clone: PC61, 1:200); CD4-BrilliantViolet 711 (clone: GK1.5, 1:200), CD4-PE-Dazzle (clone: RM4-5, 1:200); KLRG1-PE-Dazzle or -PerCP (clone: 2F1/KLRG1, 1:200); Gr1-Biotin or -FITC (clone: RB6-8C5, 1:200); Ter-119-Biotin (clone: TER-119, 1:200); CD19-FITC (clone: 6D5, 1:200); FcεRI-PE-Cy7 (clone: MAR-1, 1:200); cKit-Pacific Blue or -PerCP (clone: 2B8, 1:200); ICOS-PE (clone: C398.4A, 1:200); IgE-PE (clone: RME-1, 1:200); Streptavidin-BrilliantViolet 510 or -PE (1:100), all from Biolegend; CD5-FITC (clone: 53–7.3, 1:200); ST2-APC (clone: RMST2-2, 1:100); or CD49b – FITC (clone: DX5, 1:200) from Invitrogen, or Siglec-F-PE (clone: REA798, 1:200) from Miltenyi Biotec.

If intracellular staining was not required, cells were washed with FACS buffer and centrifuged as before and resuspended in either FACS buffer or 1 x PBS for flow cytometry analysis. For cells undergoing intranuclear transcription factor staining, cells were washed with FACS buffer, centrifuged as before and resuspended in 250 μL in FoxP3 fixation/permeabilisation buffer (ThermoFisher Scientific) for 30 min at 4°C in the dark. Cells were centrifuged as before and washed with 250 μL FoxP3 1 x permeabilisation buffer (ThermoFisher Scientific), centrifuged as before and resuspended in 1 x permeabilisation buffer containing intracellular antibodies for 30 min at 4°C in the dark, washed with 1 x permeabilisation buffer, centrifuged as before and resuspended in 1 x PBS or FACS buffer for flow cytometry analysis.

Intranuclear antibodies used: GATA3-PE (clone: TWAJ, 1:50); RORγt-APC (clone: B2D, 1:100); FoxP3-PE-Cy7 (clone: FJK-16s, 1:100), all from Invitrogen.

For intracellular cytokine staining, cells were first stimulated for 4 h at 37°C in complete RPMI media (RPMI-1640 (Gibco) supplemented with 10% FCS (Invitrogen), 100 U/ml penicillin, 100 μg/mL streptomycin (Gibco) and 2 mM L-glutamine (Gibco)) containing 1 x Cell Stimulation Cocktail plus protein transport inhibitor (Invitrogen 500x). Following stimulation, cells were centrifuged as before and resuspended ready for flow cytometry staining as described above.

Intracellular cytokine antibodies used: IL-5-PE (clone: TRFK5, 1:50) (Biolegend) and IL-13-PE-Cy7 (clone: eBio13A, 1:50) (ThermoFisher Scientific).

Samples were acquired on an LSR Fortessa (BD) and analyzed using Flowjo v10.9 (BD).

Gating strategies are shown for peritoneal lavage mast cells (live, $CD45^+ckit^+lineage^-Fc\varepsilon RI^+$) and ILC2 (live, $CD45^+KLRG1^+lineage^-$) (Figure S1); mesenteric lymph node ILC2 (live, $CD45^+lineage^-CD127^+GATA3^+ROR\gamma t^-$) or Th2 cells (live, $CD45^+CD4^+lineage^+GATA3^+Foxp3^-$) (Figure S2); perigonadal white adipose tissue ILC2 (live, $CD45^+lineage^-KLRG1^+CD25^+$) (Figure S3); and lung ILC2 (live, $CD45^+lineage^-ICOS^+CD90.2^+CD25^+CD4^-Fc\varepsilon RI^-$) (Figure S4). Lineage stains contained CD3, CD19, CD5, GR1, CD11b and CD49b. Gating strategy for eosinophils is as previously described.[13]

Serum antibody and cytokine measurements

Serum from each mouse was obtained by using injectable anesthesia overdose followed by cutting of the brachial artery. Blood was collected into a serum Microtainer SST tube (BD), mixed and left for 30 min at RT before being centrifuged at 6,000 g for 3 min to separate the red blood cells from serum and was then subsequently frozen at −70°C until required for assays.

Antigen-specific antibodies in the serum were measured by ELISA, coating Nunc 96 well plates with 1 μg/mL of the individual HpARIs or HpBARIs in 0.1 M carbonate buffer pH 9.6. Serum was firstly diluted 1:500 with ELISA block (2% BSA in 1 x TBS/0.05% Tween 20) and then serially diluted. Horseradish peroxidase-conjugated Goat anti-Mouse IgG (H + L) (BioRad) (1:2000) was used for detection, and was then developed with 1 x TMB (BioLegend) followed by a stop acid solution (1 M H_2SO_4). Plates were read at OD450 on a spectrophotometer (BMG Labtech ClarioStar). Endpoint titers were determined by identifying the reciprocal dilution of the mean plus 3 x SD of blank wells.

Cytokine measurements of serum were performed using LEGENDplex murine Th1/Th2 8 -plex Version 3 (BioLegend) as per kit instructions with data acquired using a Fortessa (BD) flow cytometer and analyzed using software by BioLegend. The limit of detection for each cytokine is marked on the graphs as a dotted line.

Murine IL-5 cytokine measurements of BAL and IL-33 response assay supernatants in were performed by ELISA using the mouse IL-5 uncoated ELISA kit (Invitrogen). Murine mast cell protease (MCPT)-4 measurements of serum and peritoneal lavage were performed by ELISA using the mouse MCPT-4 ELISA Kit (Insight Biotechnology).

IL-33 response assay

In vitro modulation of IL-33 responses was measured as described previously.[12,14] The CMT64 cell line (ECACC 10032301; RRID: CVCL_2406) was cultured in complete RPMI (as described previously) to confluency in flat 96-well plates (200 μL culture volume), in the presence or absence of HpARI1, HpARI2 or HpARI3 (100 ng/mL), and 2.5% serum from vaccinated animals as indicated. Cultures were immediately frozen on dry ice (causing cellular necrosis and release of stored IL-33), and stored at −70°C. Cells were then thawed at 37°C for 2 h and supernatants added directly to murine bone marrow cultures. 5 x 10^5 cells/well of mouse bone marrow cells were cultured in the presence of 50 μL CMT64 freeze-thaw supernatants (containing HpARIs and serum as described above), IL-2 and IL-7 (10 ng/mL final concentration each (BioLegend)). Cells were cultured for 5 days, prior to collection of supernatants for IL-5 ELISA (Invitrogen).

Histology

Dissected mouse small intestine tissues were flushed through with cold phosphate buffered saline (PBS) and inverted onto PBS-soaked wooden skewers and semi-fixed in 10% neutral buffered formalin (NBF) (Sigma) for 3–5 h, before being removed from the skewers by slicing the tissue longitudinally with a scalpel blade, allowing the tissue to be peeled off the sticks, flattened and then rolled into a 'Swiss-roll' formation. Swiss rolls were pinned with a syringe needle to retain shape before being placed into double height histology cassettes and fully submerged in 10% NBF for a further 14–19 h. The cassettes were next rinsed in PBS and transferred to 70% ethanol for 24 h prior to processing and embedding into paraffin. Cross-sections of 5 μm were cut and stained with Periodic acid-Schiff (PAS) to allow for goblet cell scoring. Slides were scanned using a Zeiss Axioscan. Goblet cell counts were performed manually in a blinded manner by 2 individuals, with the number of goblet cells counted from villus tip to villus tip, using Fiji/ImageJ open source software.

QUANTIFICATION AND STATISTICAL ANALYSIS

Data was analyzed using Prism version 10.2.3 (Graphpad). Statistical tests are detailed in figure legends: where multiple groups are compared one-way ANOVA with Dunnett's multiple comparisons test is used. Where multiple groups and timepoints are compared, two-way ANOVA and Dunnett's multiple comparisons test is used. Where two groups are compared, Mann-Whitney U test was used. All error bars indicate standard error of mean. Mouse numbers are indicated in figure legends. * = $p < 0.05$, ** = $p < 0.01$, *** = $p < 0.001$, **** = $p < 0.0001$, ns = $p > 0.05$.

Cell Reports, Volume *44*

Supplemental information

Vaccination against helminth IL-33 modulators permits immune-mediated parasite ejection

Danielle J. Smyth, Suzanne H. Hodge, Nicole W.P. Ong, Josh Richards, Florent Colomb, Samuele Di Carmine, Vivien Shek, Tania Frangova, Marta Campillo Poveda, Rick M. Maizels, and Henry J. McSorley

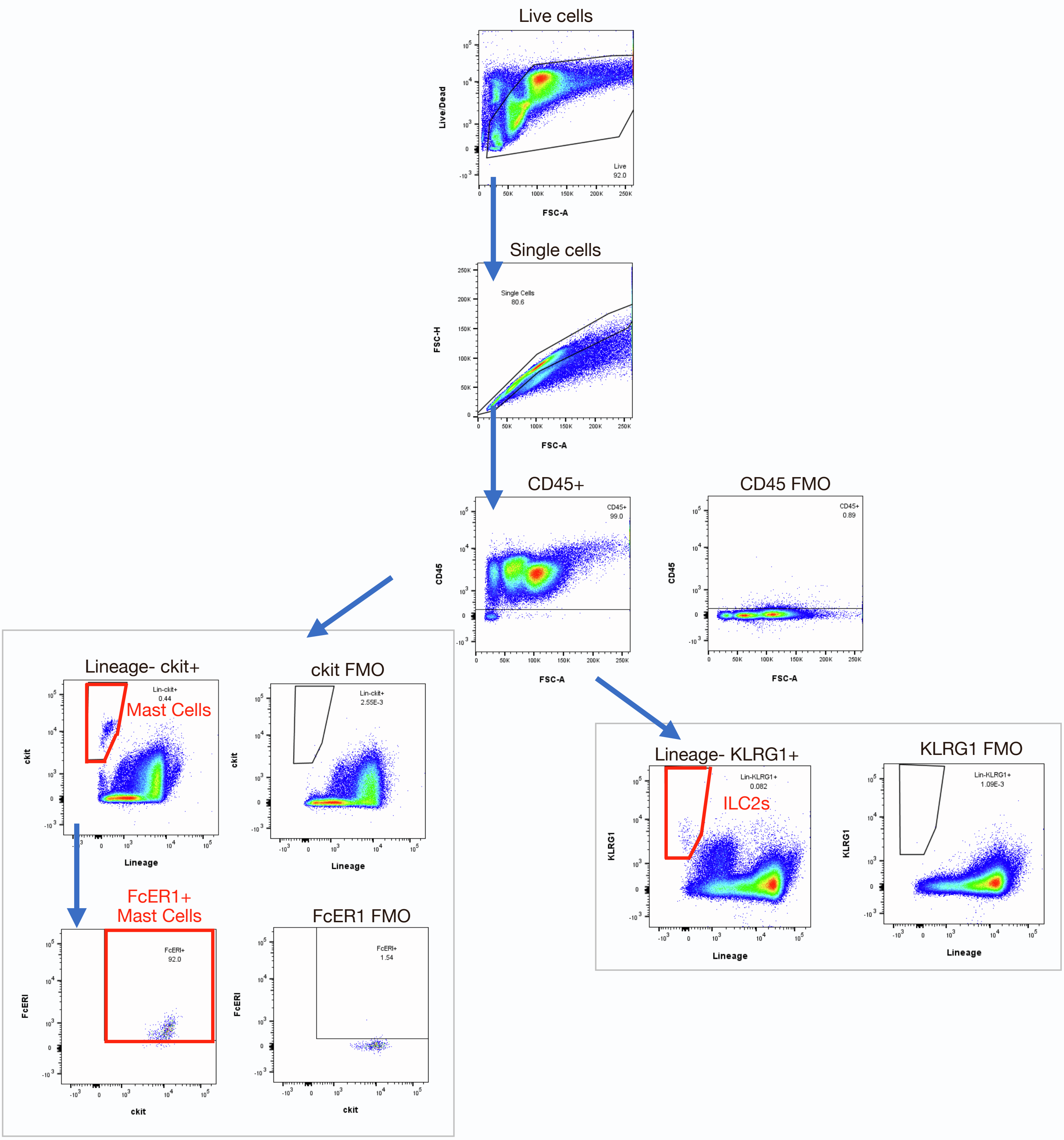

Supplementary Figure 1: Peritoneal lavage mast cell and ILC2 flow cytometry gating strategy

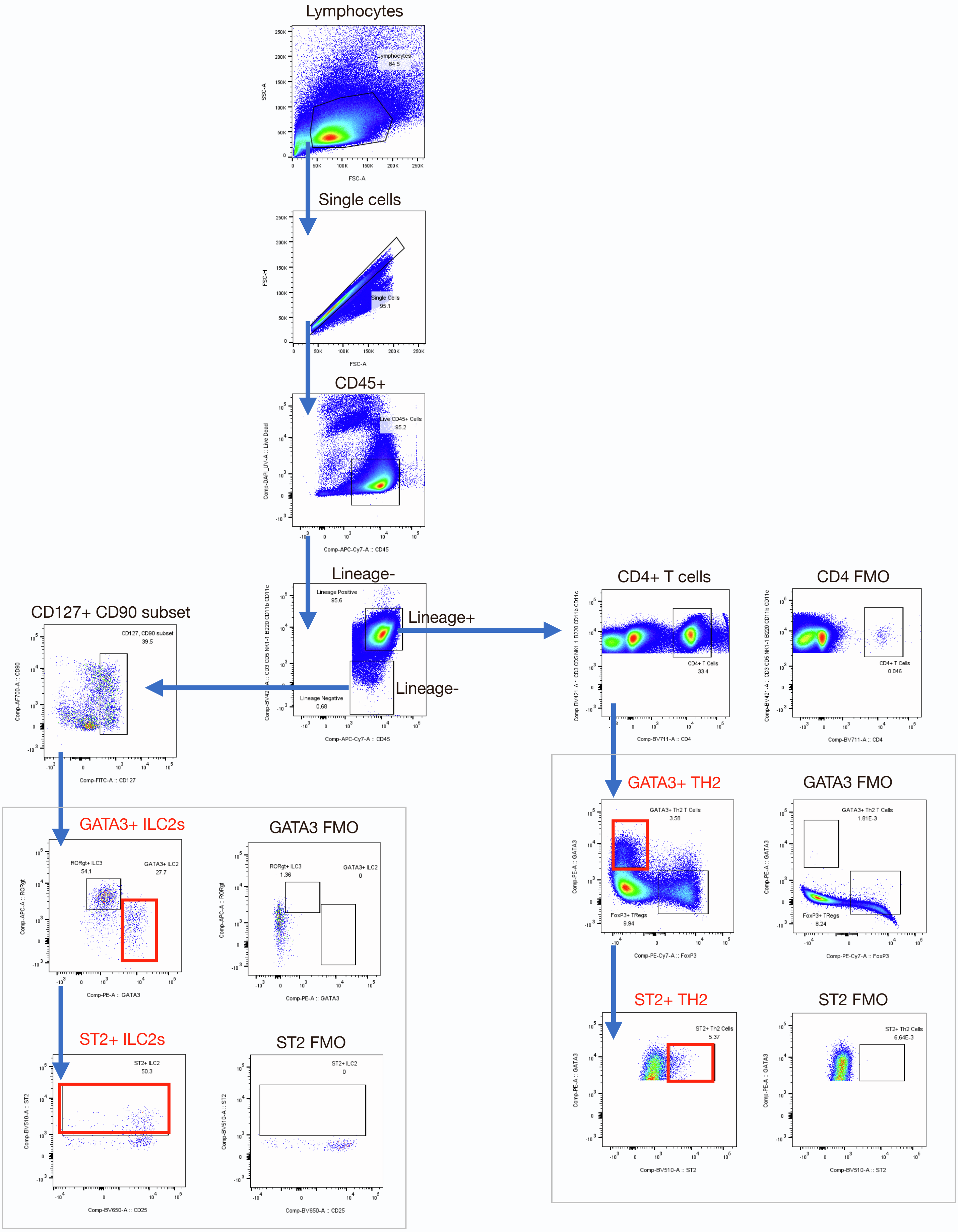

Supplementary Figure 2: Mesenteric lymph node ILC2 and CD4+ Th2 cell flow cytometry gating strategy

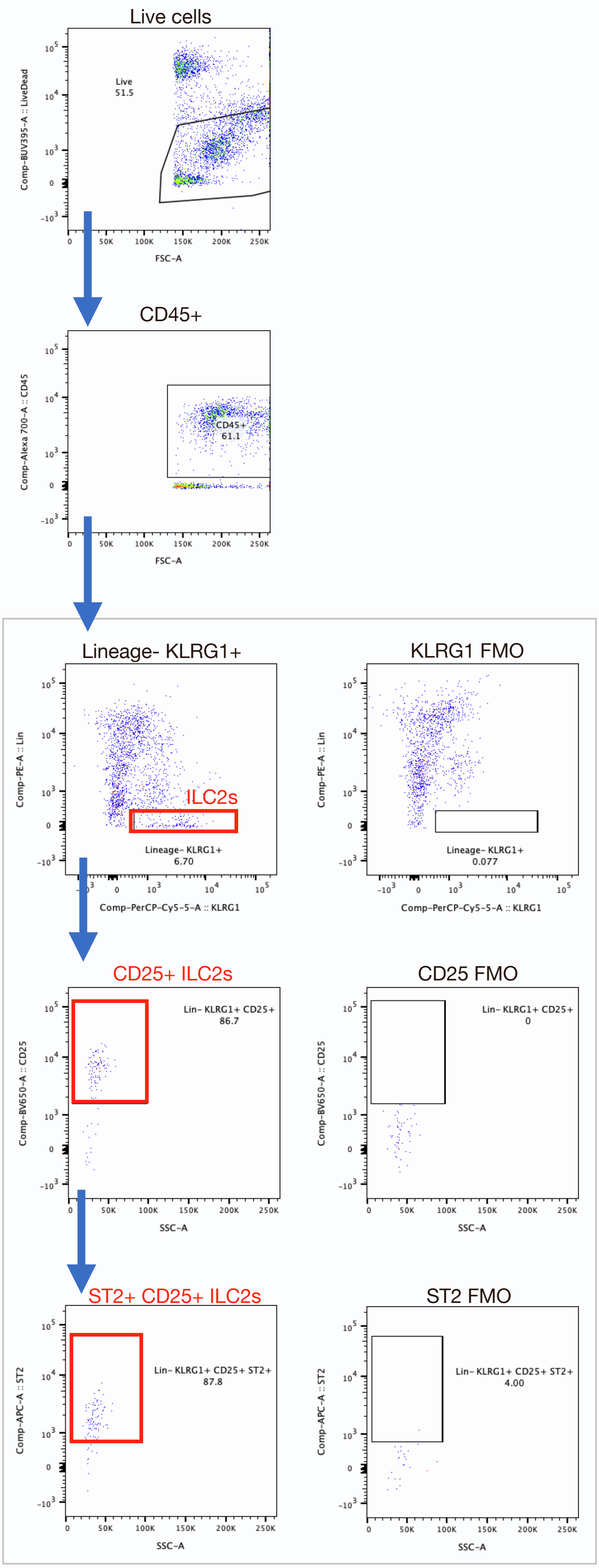

Supplementary Figure 3: pgWAT ILC2 flow cytometry gating strategy

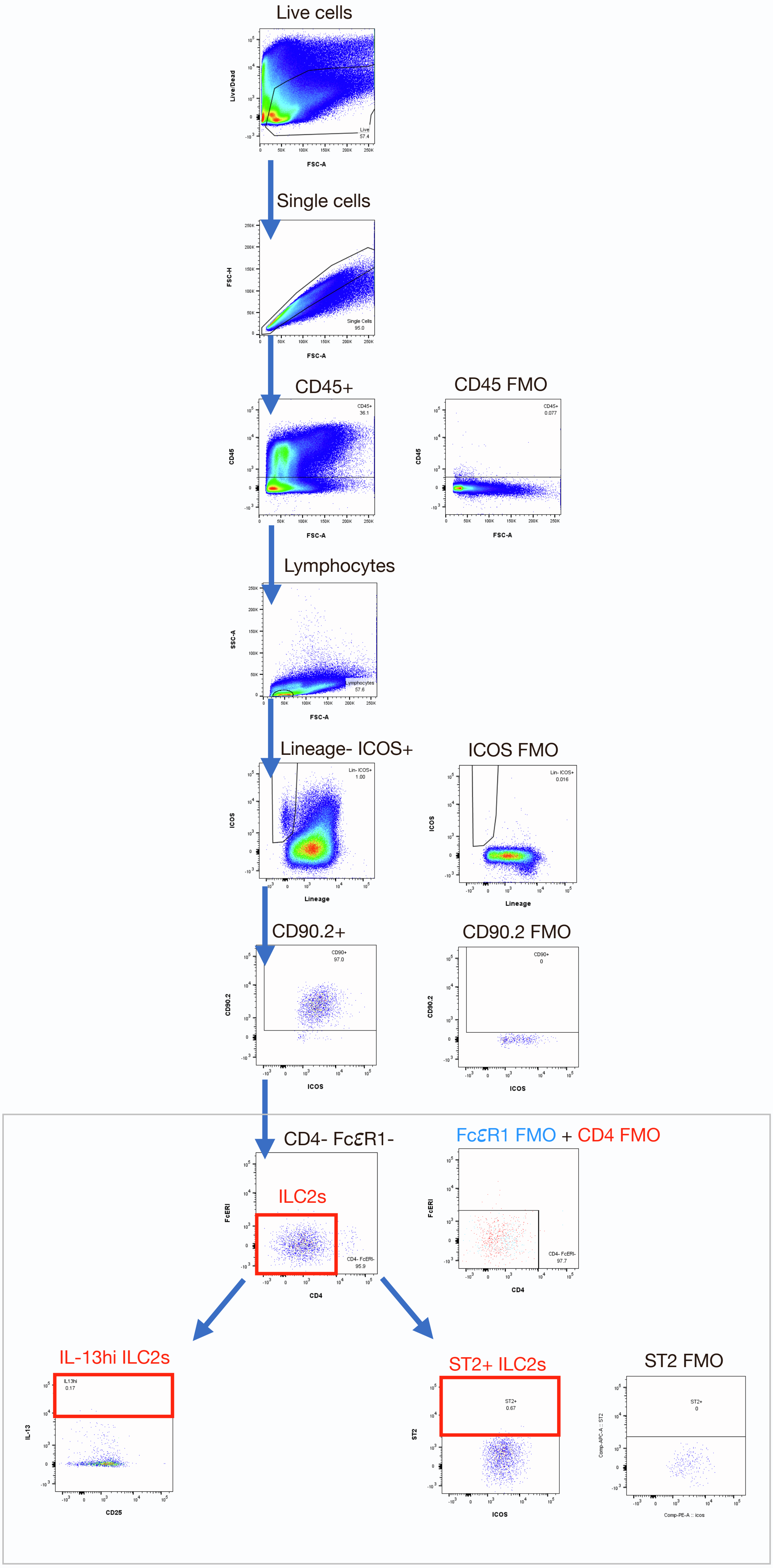

Supplementary Figure 4: Lung ILC2 flow cytometry gating strategy

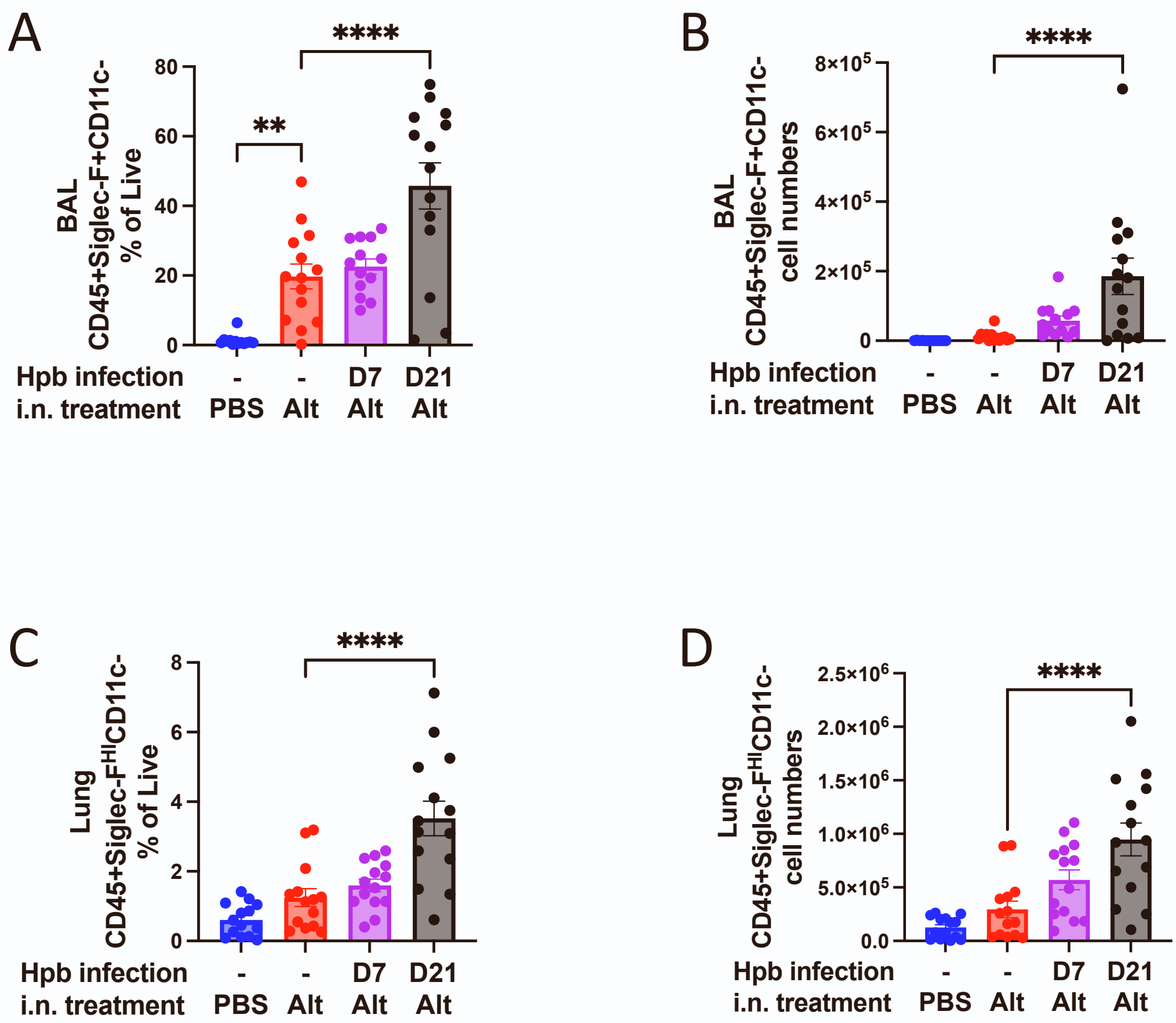

Supplementary Figure 5: Eosinophil responses in *Hpb*-infected mice treated with Alternaria allergen.
IL-13+/eGFP mice (mixed sexes; 8-15 weeks old) were infected with 200 L3 *Hpb* 7 or 21 days prior to intranasal administration of Alternaria extract, then culled 24 h after Alternaria administration, as described in Figure 1. Eosinophils in the BAL (A, B) and lung tissue (C, D) were identified by flow cytometry. Eosinophil % of live cells is shown in A and C, while total eosinophil numbers are shown in B and D. Data pooled from 3 repeat experiments, for a total of 13-14 mice per group. Analysed by one-way ANOVA followed by Šídák's multiple comparisons test, comparing groups that share a variable. Unless otherwise stated, differences are not significant. * = $p<0.05$, ** = $p<0.01$, *** = $p<0.001$, **** = $p<0.0001$.

A

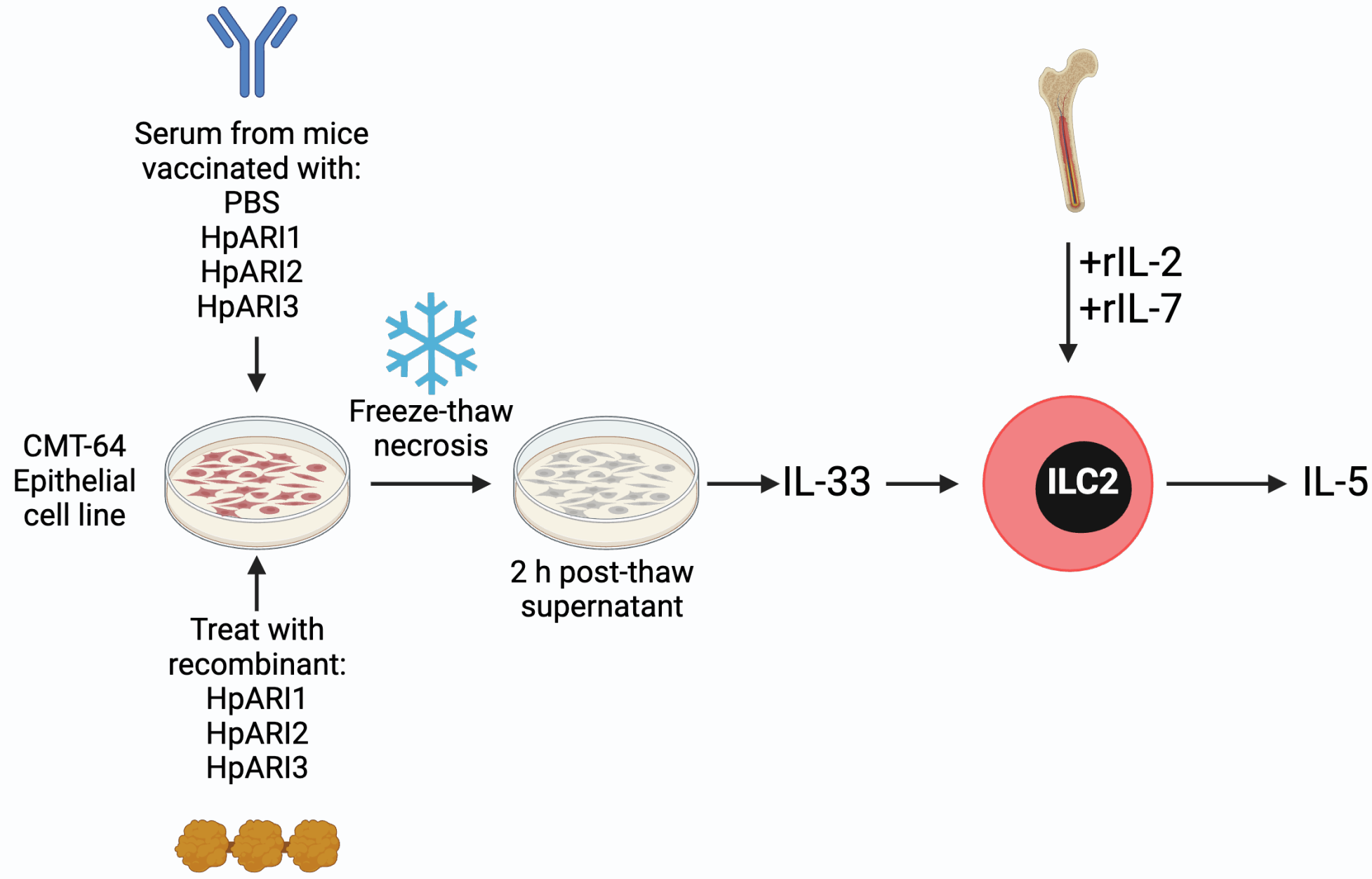

Supplementary Figure 6: Vaccination serum to abrogate effects of HpARI1, HpARI2 or HpARI3 in vitro
Experimental setup of IL-33 response assay. CMT-64 cells were freeze-thawed to induce necrosis and IL-33 release, in the presence or absence of HpARIs, and serum from mice at day 7 of *Hpb* infection, after vaccination with HpARIs. Supernatants containing IL-33 were taken from necrotic CMT-64 cultures 2 hours after thaw, and added to female mouse bone marrow cells in the presence of IL-2 and IL-7 (10 ng/ml each). Bone marrow cells were cultured for 5 days, and IL-5 in supernatants measured by ELISA.

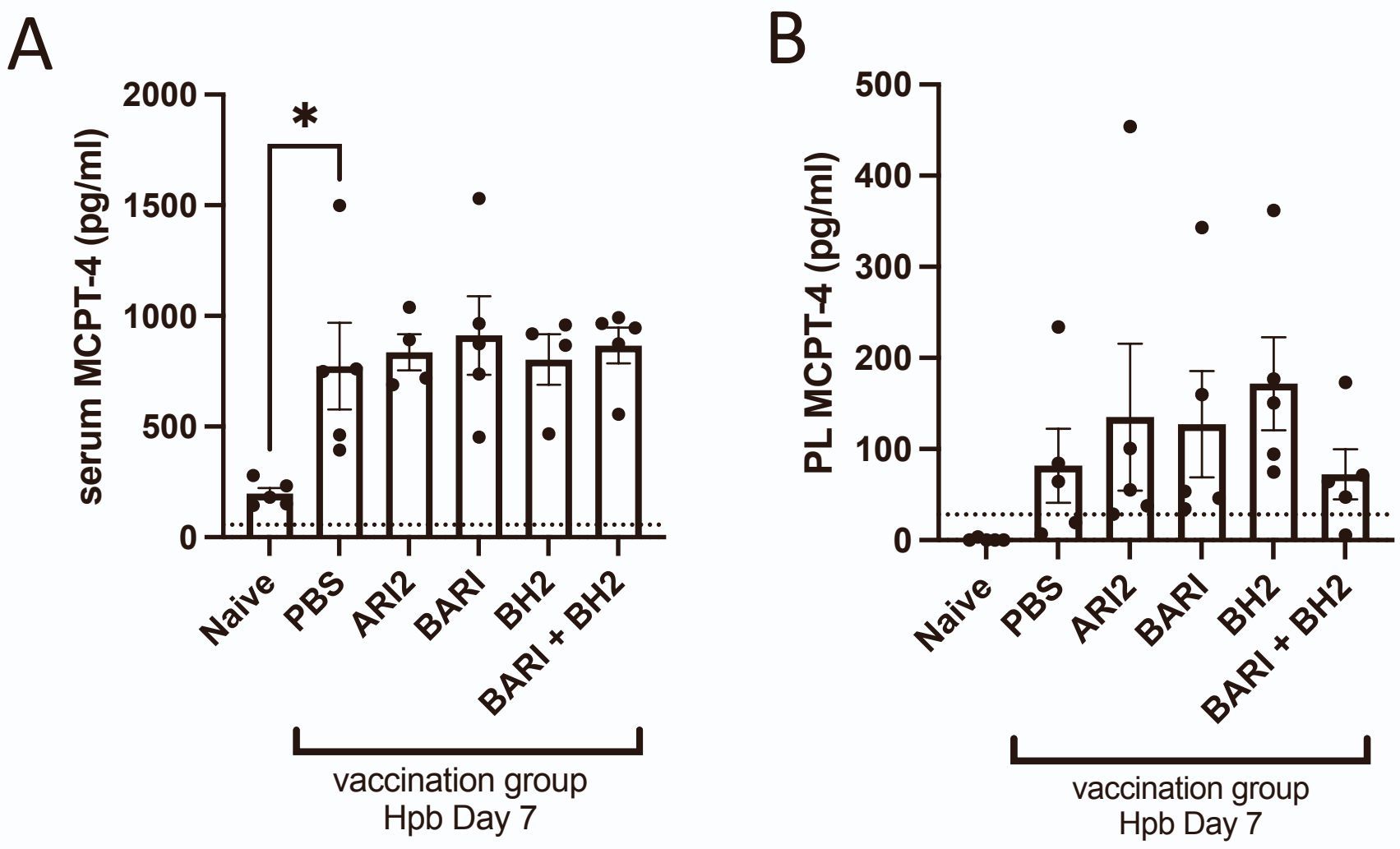

Supplementary Figure 7: Vaccination does not affect mast cell degranulation markers

MCPT-4 levels were measured by ELISA in the serum (A) and peritoneal lavage (B) of vaccinated mice at day 7 of infection.

Data representative of 2 repeat experiments. Female C57BL/6J mice aged 6-12 weeks were used, 5 mice per group. Analysed by one-way ANOVA followed by Dunnett's multiple comparisons test, comparing all groups to PBS-vaccinated infected mice. Dotted line on y axis indicates limit of detection. Unless otherwise stated, differences are not significant. * = $p<0.05$.

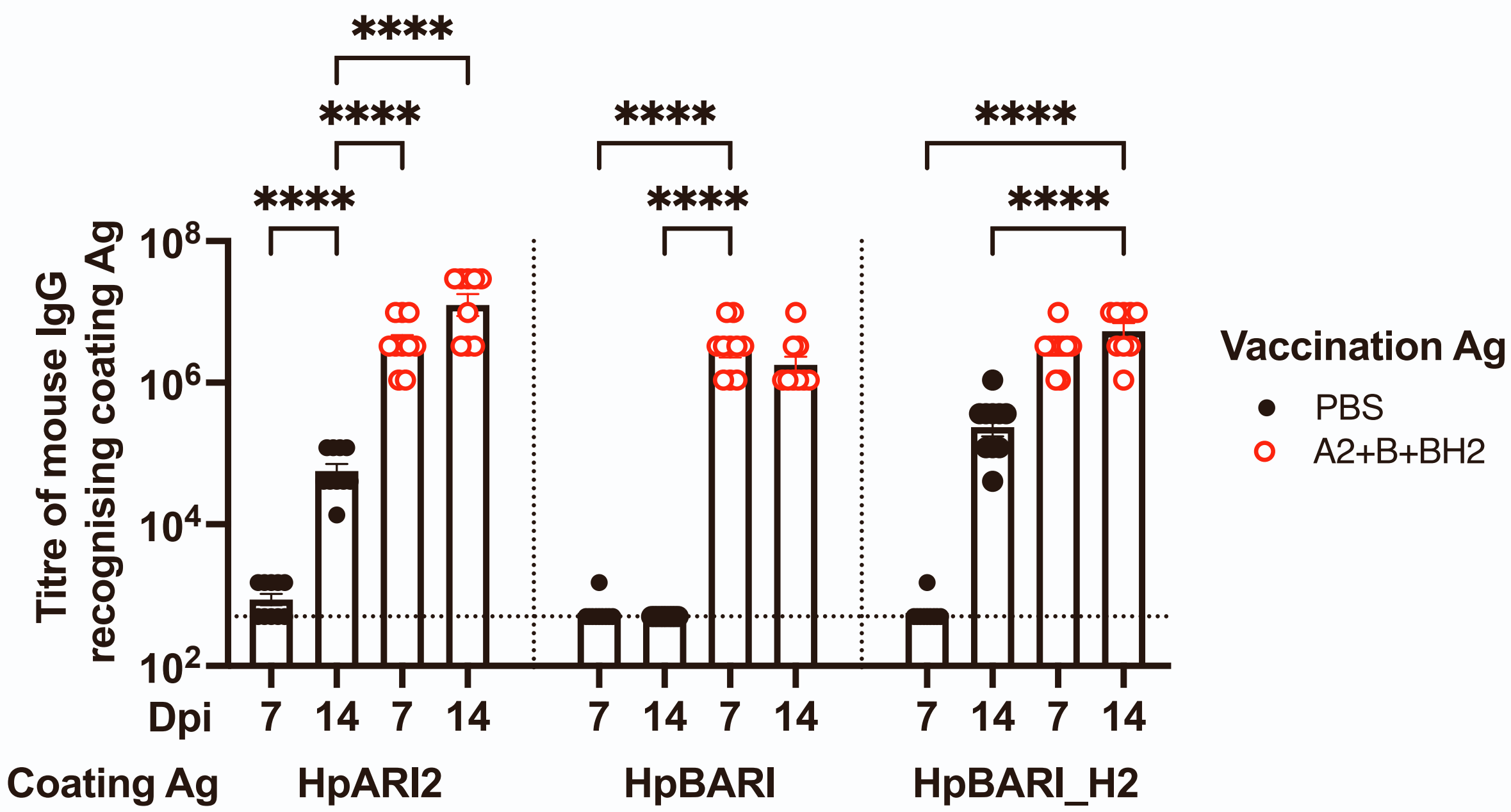

Supplementary Figure 8: Serum antibody responses to HpARI2+HpBARI+HpBARI_Hom2 vaccination

HpARI2, HpBARI and HpBARI_Hom2-specific serum IgG titres after HpARI2+HpBARI+HpBARI_Hom2 (A2+B+BH2) vaccination, at day 7 or 14 of *Hpb* infection. Data pooled from 2 repeat experiments. Female C57BL/6J mice aged 6-12 weeks were used. Data in Day 7 post infection is pooled from 2 repeat experiments, for a total of n=10 per group. Data in Day 14 post infection is pooled from 2 repeat experiments for a total group size of n=10 for PBS vaccination and n=9 for A2+B+BH2 vaccination. Analysed by one-way ANOVA followed by Dunnett's multiple comparisons test. Unless otherwise stated, differences are not significant. * = p<0.05, ** = p<0.01, *** = p<0.001, **** = p<0.0001.
