# Supplementary material for: Vaccination against helminth IL-33 modulators permits immune-mediated parasite ejection
Source: Cell Rep. Author manuscript; Available in PMC 2026 Aug 15. (PMC7619366; doi:10.1016/j.celrep.2025.115721)
Supplement: Supplementary file 2 [file EMS217148-supplement-Document_S2__Article_plus_supplemental_information_.pdf]

Cell Reports, Volume *44*

Supplemental information

Vaccination against helminth IL-33 modulators permits immune-mediated parasite ejection

Danielle J. Smyth, Suzanne H. Hodge, Nicole W.P. Ong, Josh Richards, Florent Colomb, Samuele Di Carmine, Vivien Shek, Tania Frangova, Marta Campillo Poveda, Rick M. Maizels, and Henry J. McSorley

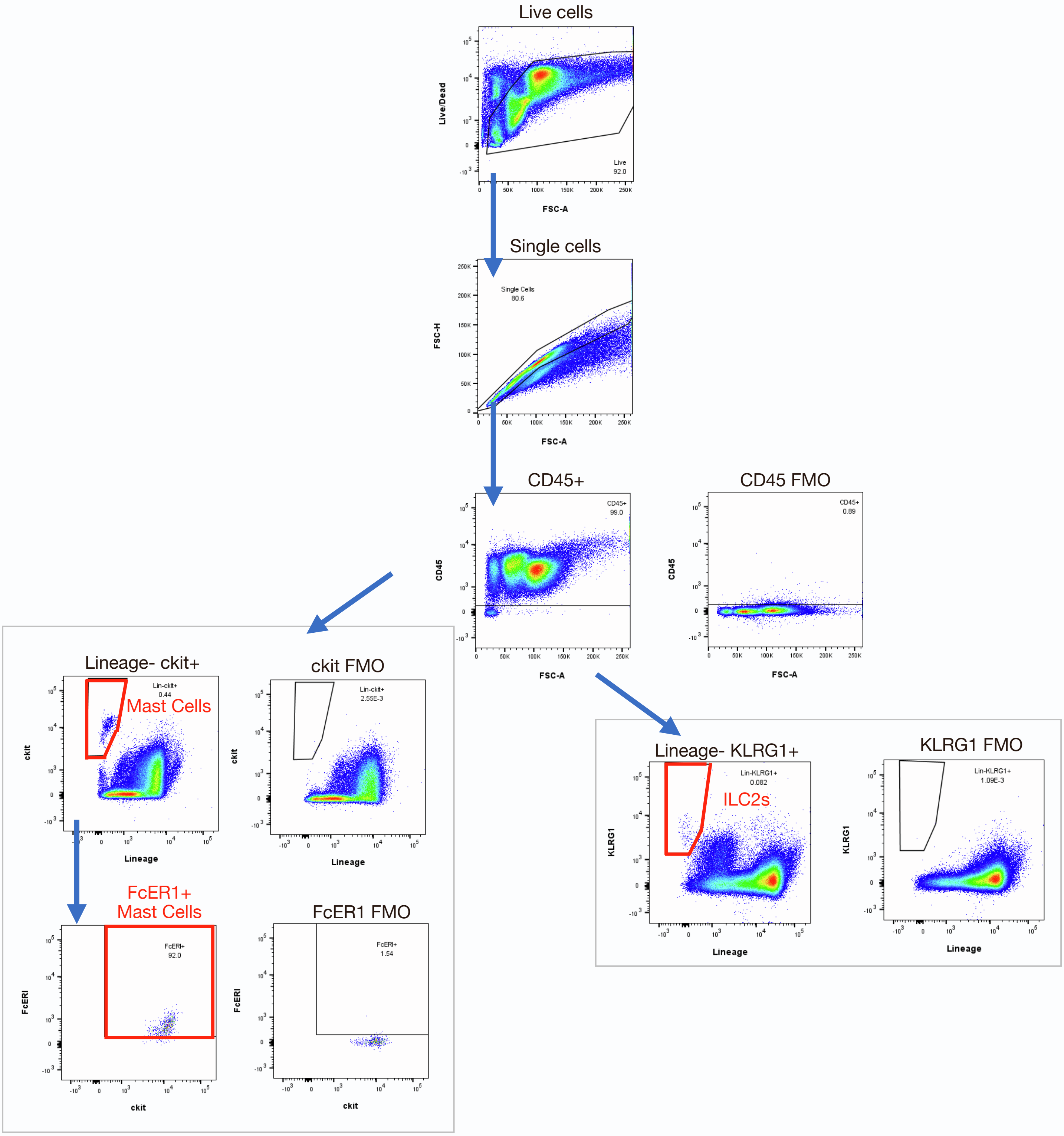

Supplementary Figure 1: Peritoneal lavage mast cell and ILC2 flow cytometry gating strategy

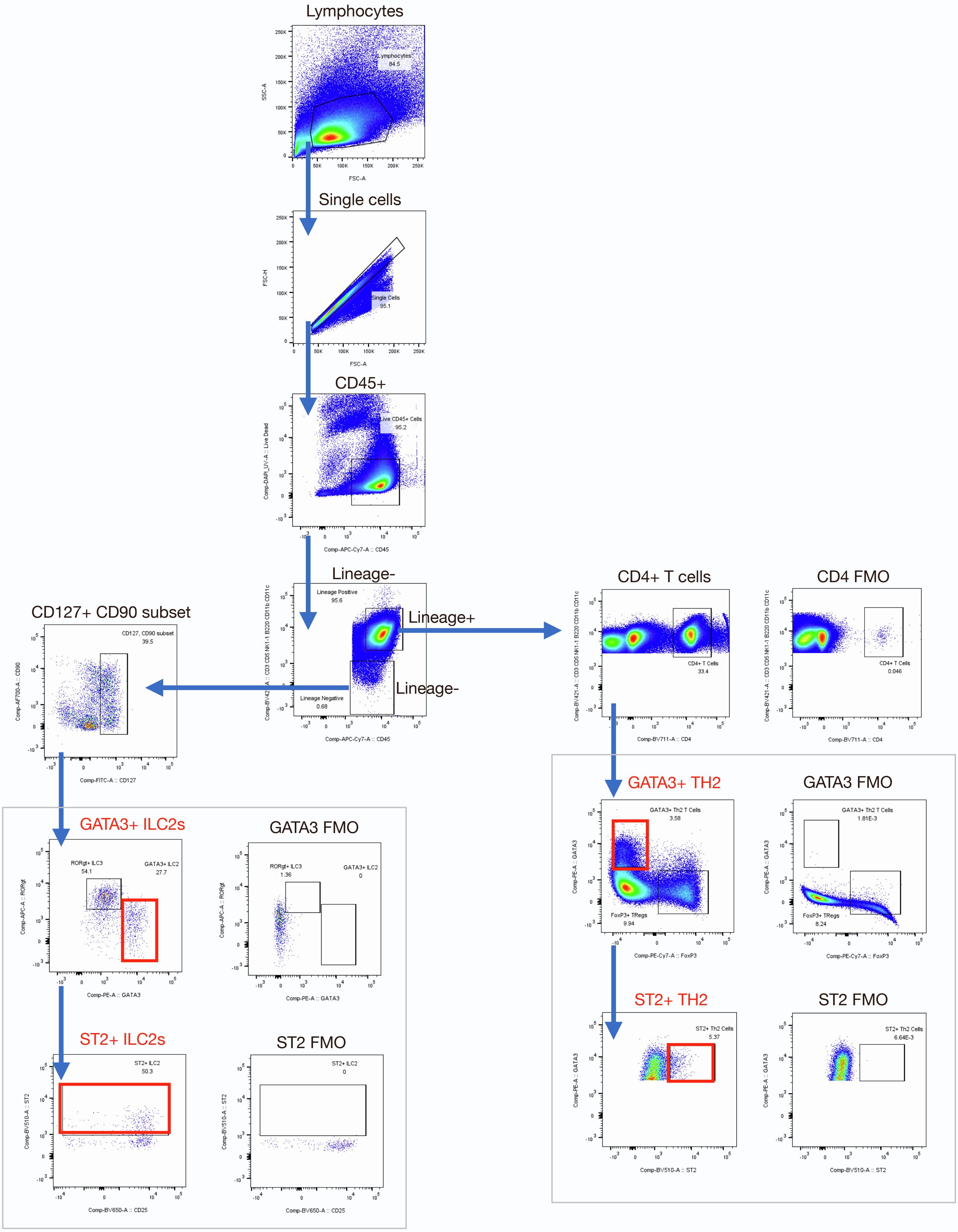

Supplementary Figure 2: Mesenteric lymph node ILC2 and CD4+ Th2 cell flow cytometry gating strategy

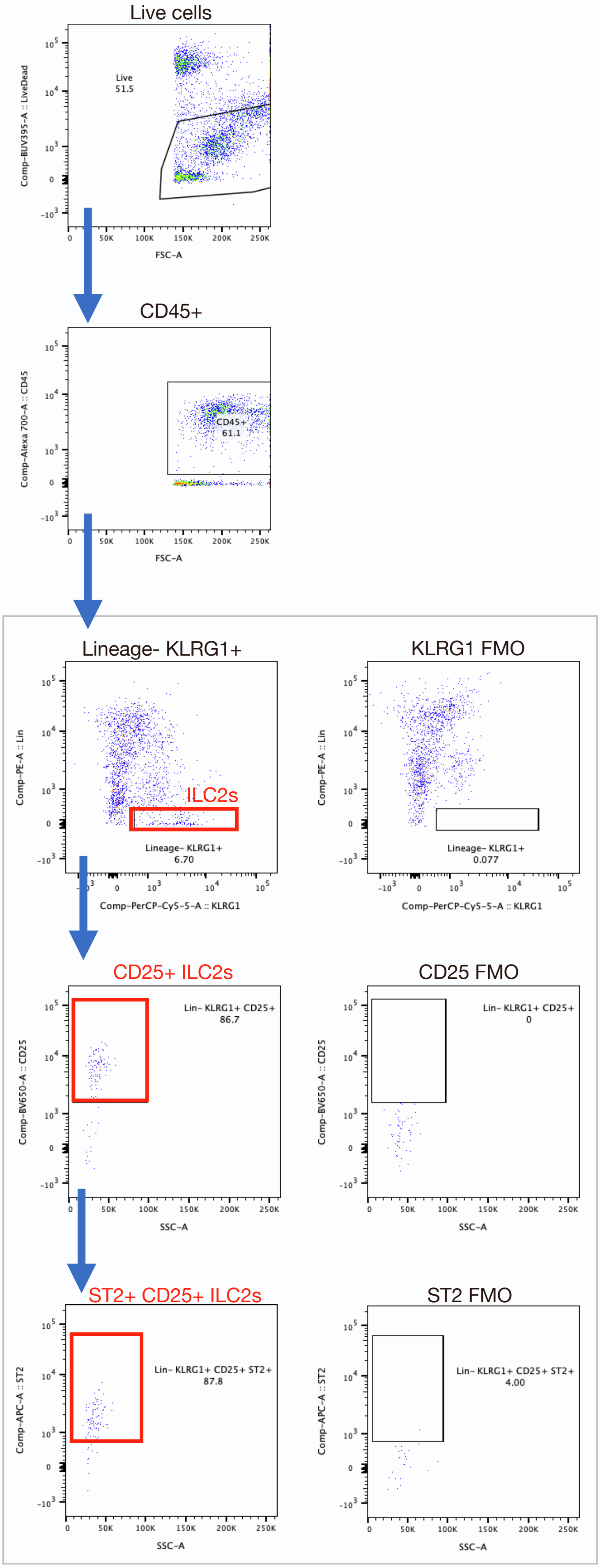

Supplementary Figure 3: pgWAT ILC2 flow cytometry gating strategy

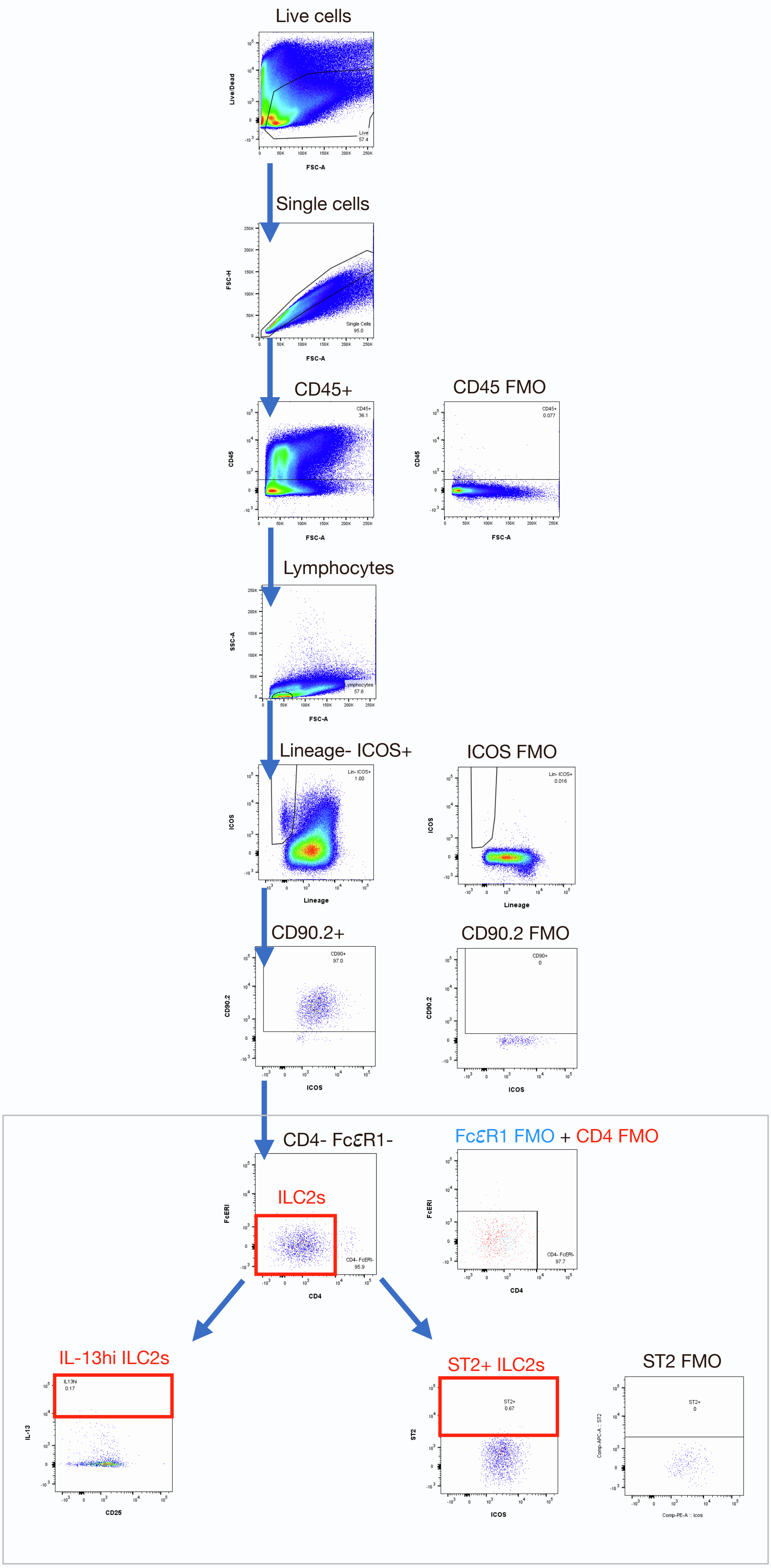

Supplementary Figure 4: Lung ILC2 flow cytometry gating strategy

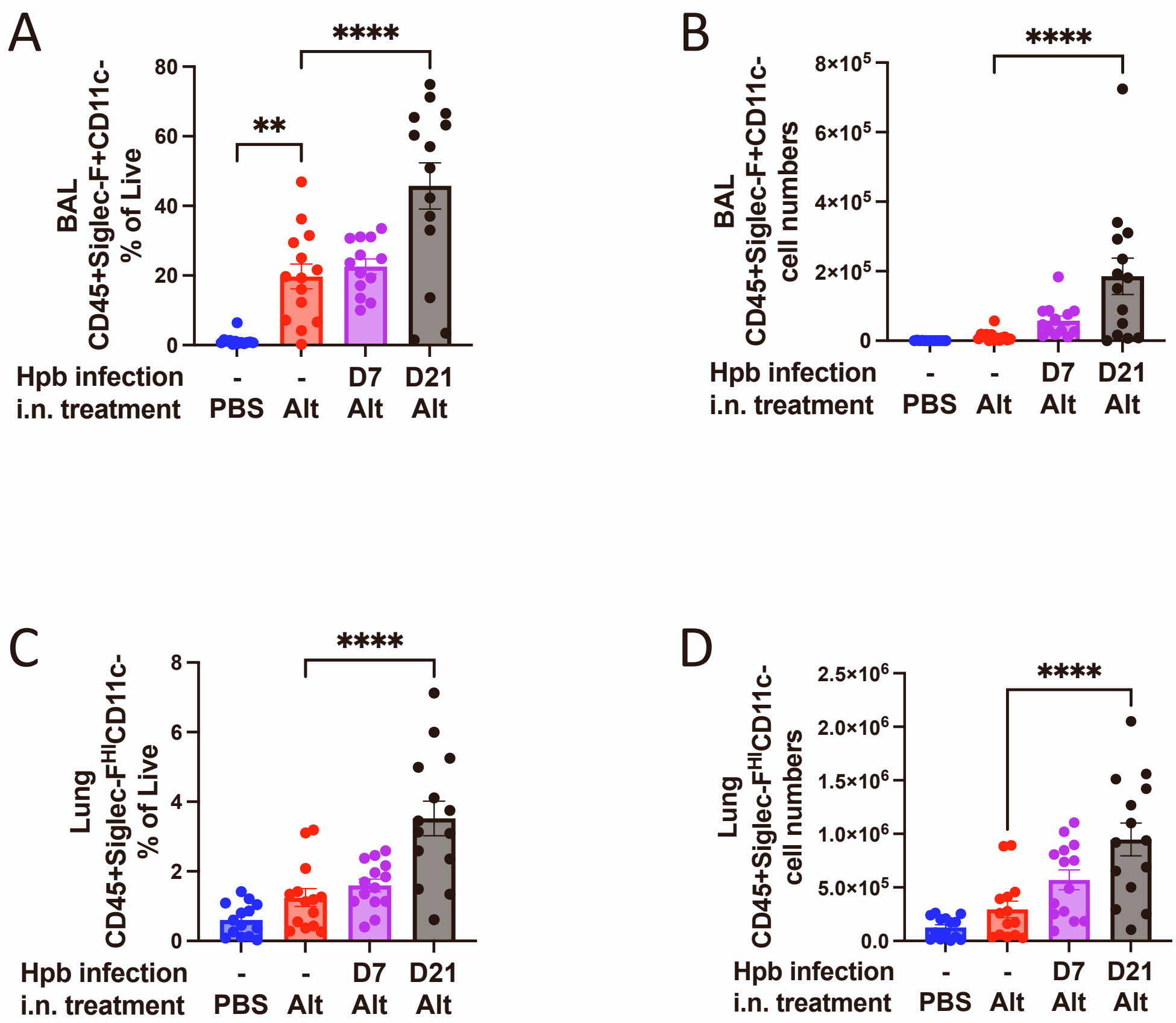

Supplementary Figure 5: Eosinophil responses in *Hpb*-infected mice treated with Alternaria allergen.
IL-13+/eGFP mice (mixed sexes; 8-15 weeks old) were infected with 200 L3 *Hpb* 7 or 21 days prior to intranasal administration of Alternaria extract, then culled 24 h after Alternaria administration, as described in Figure 1. Eosinophils in the BAL (A, B) and lung tissue (C, D) were identified by flow cytometry. Eosinophil % of live cells is shown in A and C, while total eosinophil numbers are shown in B and D. Data pooled from 3 repeat experiments, for a total of 13-14 mice per group. Analysed by one-way ANOVA followed by Šídák's multiple comparisons test, comparing groups that share a variable. Unless otherwise stated, differences are not significant. * = $p<0.05$, ** = $p<0.01$, *** = $p<0.001$, **** = $p<0.0001$.

A

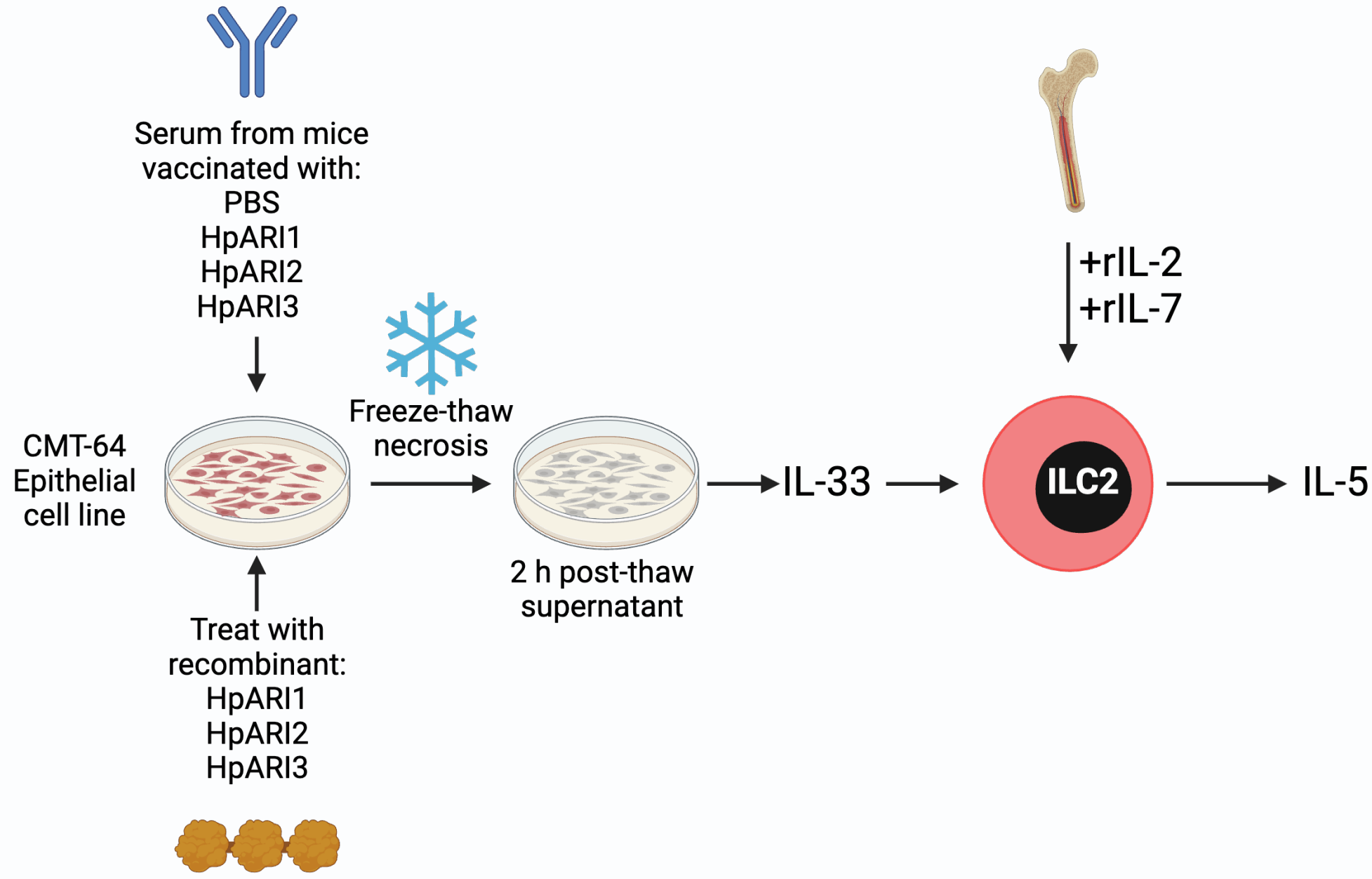

Supplementary Figure 6: Vaccination serum to abrogate effects of HpARI1, HpARI2 or HpARI3 in vitro

Experimental setup of IL-33 response assay. CMT-64 cells were freeze-thawed to induce necrosis and IL-33 release, in the presence or absence of HpARIs, and serum from mice at day 7 of *Hpb* infection, after vaccination with HpARIs. Supernatants containing IL-33 were taken from necrotic CMT-64 cultures 2 hours after thaw, and added to female mouse bone marrow cells in the presence of IL-2 and IL-7 (10 ng/ml each). Bone marrow cells were cultured for 5 days, and IL-5 in supernatants measured by ELISA.

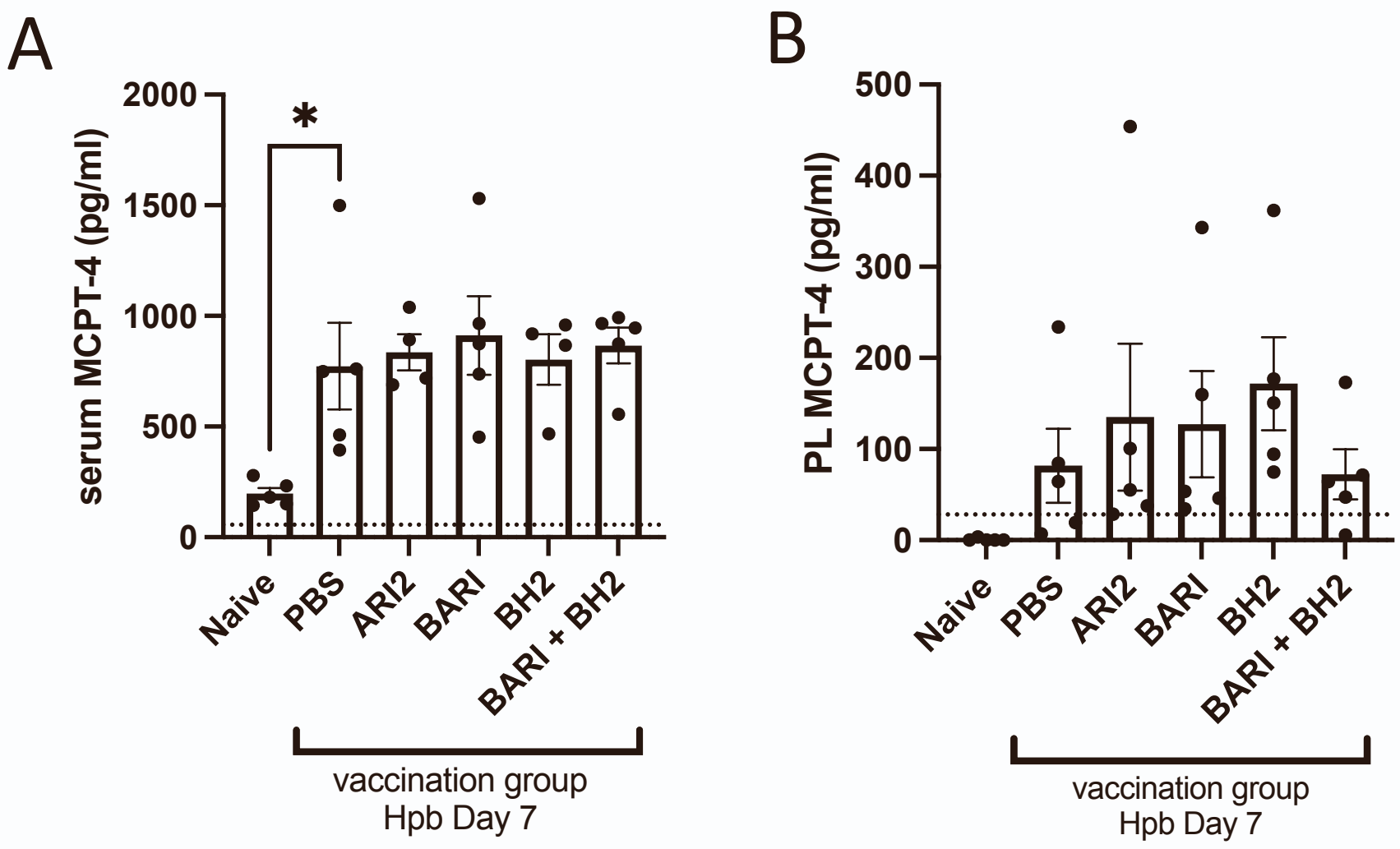

Supplementary Figure 7: Vaccination does not affect mast cell degranulation markers

MCPT-4 levels were measured by ELISA in the serum (A) and peritoneal lavage (B) of vaccinated mice at day 7 of infection.

Data representative of 2 repeat experiments. Female C57BL/6J mice aged 6-12 weeks were used, 5 mice per group. Analysed by one-way ANOVA followed by Dunnett's multiple comparisons test, comparing all groups to PBS-vaccinated infected mice. Dotted line on y axis indicates limit of detection. Unless otherwise stated, differences are not significant. * = $p<0.05$.

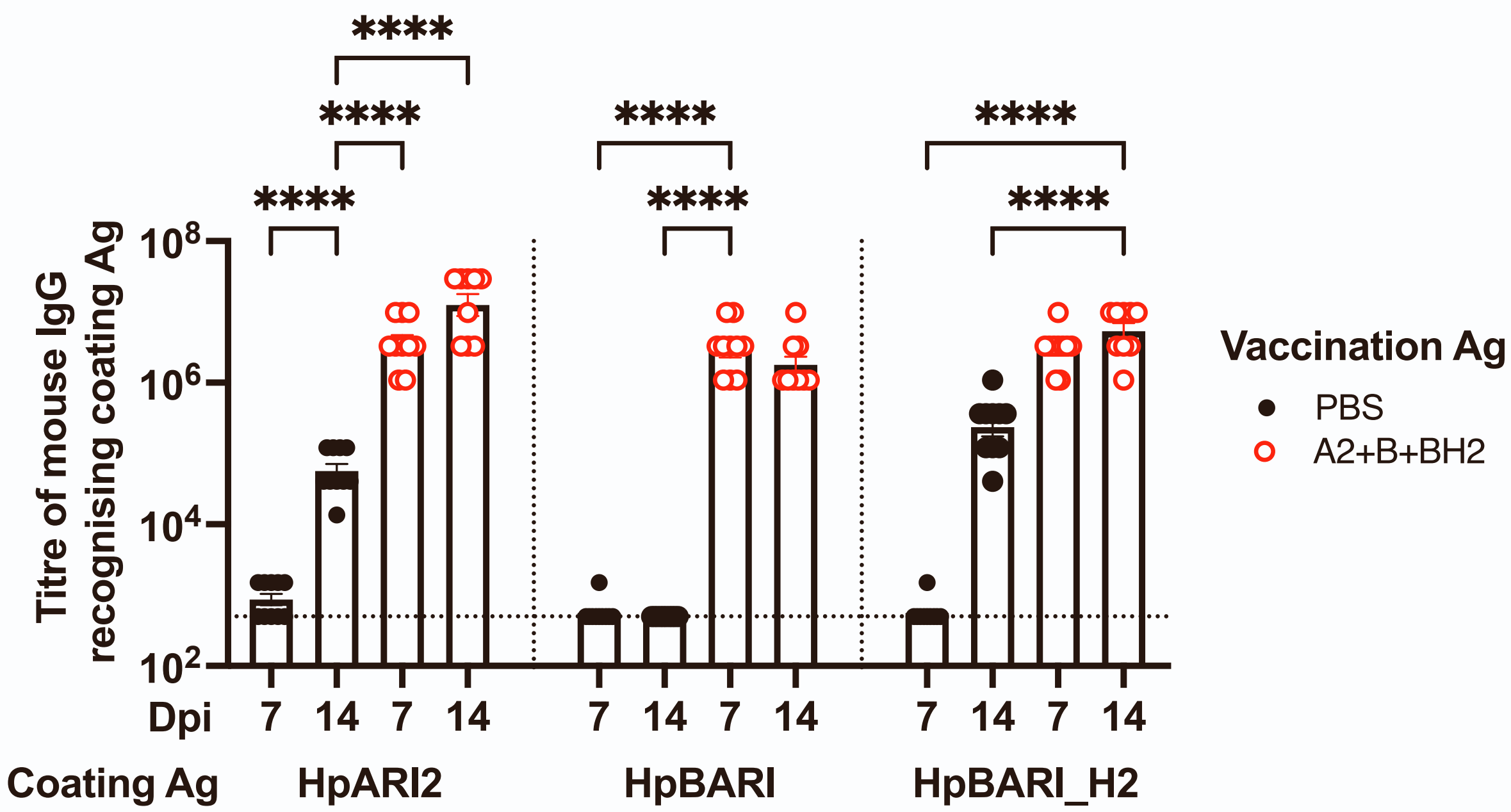

Supplementary Figure 8: Serum antibody responses to HpARI2+HpBARI+HpBARI_Hom2 vaccination

HpARI2, HpBARI and HpBARI_Hom2-specific serum IgG titres after HpARI2+HpBARI+HpBARI_Hom2 (A2+B+BH2) vaccination, at day 7 or 14 of *Hpb* infection. Data pooled from 2 repeat experiments. Female C57BL/6J mice aged 6-12 weeks were used. Data in Day 7 post infection is pooled from 2 repeat experiments, for a total of n=10 per group. Data in Day 14 post infection is pooled from 2 repeat experiments for a total group size of n=10 for PBS vaccination and n=9 for A2+B+BH2 vaccination. Analysed by one-way ANOVA followed by Dunnett's multiple comparisons test. Unless otherwise stated, differences are not significant. * = p<0.05, ** = p<0.01, *** = p<0.001, **** = p<0.0001.
